# Revealing the mechanistic correlation between volatile flavor retention and cooking oil fume reduction in stir-fried pork belly

**DOI:** 10.1016/j.fochx.2026.104547

**Published:** 2026-09-30

**Authors:** Jingnan Lu, Honglie Ding, Jiajin Sun, Lizhang Wu, Manzi Hu, Kang Xu, Huimin Wang, Xingguang Chen, Kezhou Cai, Baocai Xu

**Affiliations:** a Key Laboratory for Animal Food Green Manufacturing and Resource Mining of Anhui Province, Hefei University of Technology, Hefei 230009, China; b School of Food and Bioengineering, Hefei University of Technology, Hefei 230009, China; c Graduate Institute of Biomedical Engineering, National Chung Hsing University, Taichung City 402, Taiwan, China

**Keywords:** Cooking oil fumes, Pork belly, Flavor, Intelligent stir-frying machine, Aldehydes and ketones

## Abstract

The relationship between cooking oil fumes (COFs) control and meat flavor retention remains inadequately elucidated. In this study, an intelligent stir-frying system was employed to systematically investigate the effects of various fume-exhaust configurations on the flavor profile of pork belly and concomitant emissions of COFs. In the closed-exhaust group (CC), a total of 39 volatile compounds were identified, among which 3-methylbutanal, 2-methylbutanal, hexanal, and 2,5-dimethylpyrazine were identified as the key aroma-active components. Meanwhile, the CC group exhibited the most pronounced umami and saltiness characteristics. Nevertheless, the total airborne concentration of aldehydes and ketones in the CC group reached 4868.89 μg/m^3^, approximately 6.5 times that recorded in the open-exhaust group (OO). Network analysis revealed that fume-derived aldehydes and ketones exhibited both synergistic and competitive interactions with meat-derived volatile flavor compounds. Collectively, these findings provide a basis for achieving an optimal balance between flavor enhancement and effective COFs mitigation.

## Highlights

• The four exhaust modes of the intelligent stir-frying system allow precise control of cooking fume exposure.
• Aldehyde and ketone emissions in CC mode are approximately 6.5 times higher than those in OO mode.
• The exhaust mode regulates the volatile flavor characteristics of pork belly.
• A correlation network was established between carbonyl compounds from cooking fumes and volatile compounds from the meat.

## Graphical abstract

**Figure:**
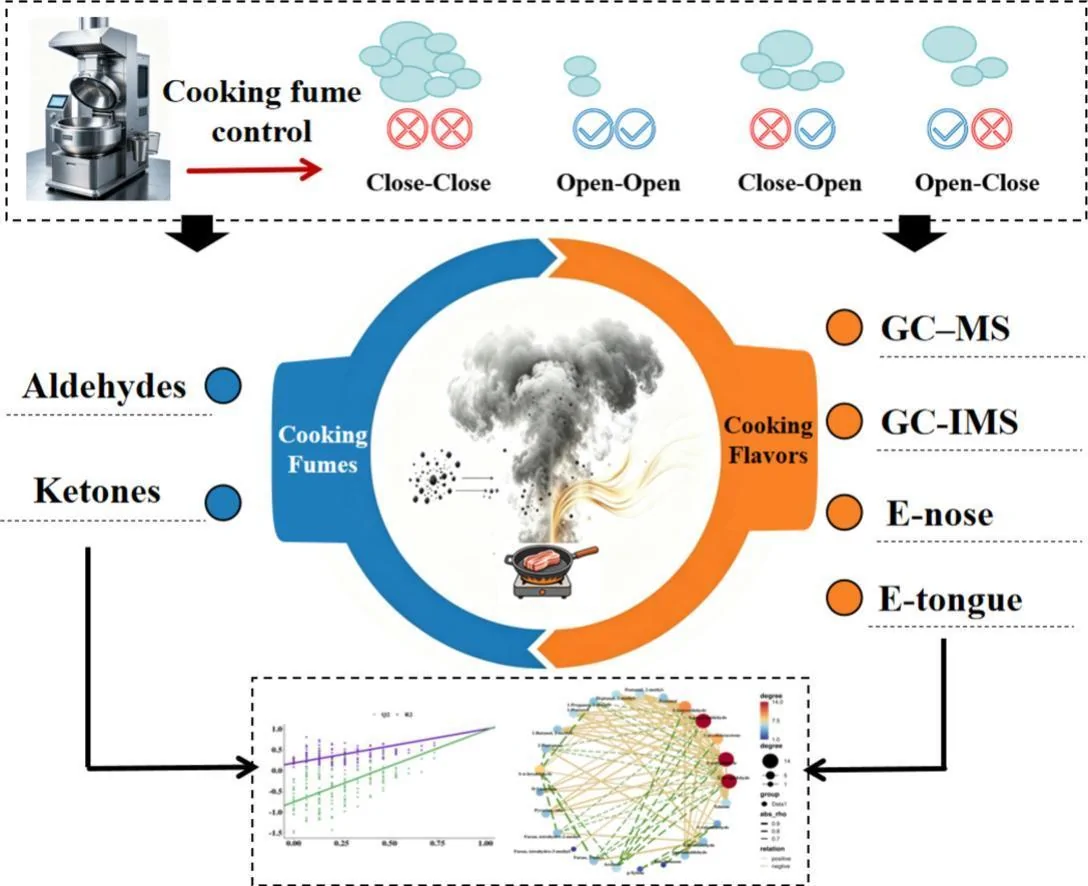

## 1 Introduction

Stir-frying is a hallmark of Chinese cuisine and is widely practiced because of its efficient heat transfer, which enables rapid cooking and flavor development (Wang, Nie, et al., 2023). Nevertheless, the high temperatures inherent in stir-frying inevitably generate substantial quantities of cooking oil fumes (COFs) (Cummings et al., 2023; Jiao et al., 2025). COFs are complex aerosols generated through the volatilization of lipid-soluble food constituents, the thermal decomposition of reaction intermediates, and the release of gaseous oxidation products. They contain particulate matter and diverse organic compounds, including alcohols, aldehydes, ketones, and polycyclic aromatic hydrocarbons (PAHs) (Liu et al., 2019; Reyes-Villegas et al., 2018; Sankhyan et al., 2021). Epidemiological and toxicological evidence indicates that chronic exposure to COFs is associated with an elevated risk of respiratory disorders, cardiovascular diseases, and lung cancer (Lv et al., 2025). Consequently, effectively mitigating COF emissions has become a pressing challenge in modern food processing industries.

Pork belly is widely appreciated for its characteristic distribution of intramuscular and subcutaneous fat, which contributes to its rich and full-bodied flavor after thermal processing (Jo et al., 2022). This flavor generation is largely attributed to the high content of unsaturated fatty acids in the adipose tissue, which are susceptible to thermally induced oxidation and free-radical cleavage, leading to the formation of characteristic volatile carbonyl compounds, particularly aldehydes (Wang, Wang, et al., 2023). These compounds are major contributors to the distinctive lard-like aroma and meaty notes of cooked pork. Notably, the same classes of carbonyl species are also prominent chemical components of COFs (Liu et al., 2022). This overlap suggests that flavor-active volatiles and COF constituents share common thermochemical origins (Scianò et al., 2025; Zhu, Guo, et al., 2021). The fume-exhaust configuration may alter the concentration of volatile compounds within the cooking chamber, thereby shifting the competitive balance between lipid oxidation and the Maillard reaction. These changes can influence the formation and retention of characteristic flavor compounds, ultimately modifying the final flavor profile of the cooked product. However, experimental evidence remains limited regarding whether fume control causes an overall loss of flavor compounds or selectively alters the characteristic flavor attributes.

In this study, pork belly was selected as the experimental food matrix. Different COF emission conditions were established by adjusting the chamber aperture via the integrated fume-control system of an intelligent stir-frying machine. The effects of these COF emission conditions on the composition, abundance, and overall profile of volatile flavor compounds in stir-fried pork belly were systematically evaluated. Concurrently, the emissions of aldehydes and ketones in the cooking environment were determined. Correlation analysis was performed to quantify the associations of fume-derived aldehydes and ketones with key meat-derived flavor compounds. This study provides a theoretical basis for optimizing fume-control strategies in intelligent cooking appliances while preserving the desirable flavor attributes of cooked foods.

## 2 Materials and methods

### 2.1 Materials and reagents

Fresh pork belly was procured from a local supermarket in Hefei, Anhui Province, China. Peanut oil and edible salt were supplied by Shandong Luhua Group Co., Ltd. (Shandong, China) and China National Salt Industry Group Co., Ltd. (Beijing, China). Monosodium glutamate, soy sauce, and cooking wine were obtained from Shanghai Totole Food Co., Ltd. (Shanghai, China), Foshan Haitian Flavoring & Food Co., Ltd. (Foshan, Guangdong, China), and Jiangsu Hengshun Vinegar Industry Co., Ltd. (Zhenjiang, Jiangsu, China), respectively. Tridecanoin (C11:0), 1,1,3,3-tetraethoxypropane, and a 13-component aldehyde and ketone standard mixture were purchased from ANPEL Laboratory Technologies, Inc. (Shanghai, China). A 37-component fatty acid methyl ester (FAME) standard mixture and 15% boron trifluoride-methanol solution were obtained from the same supplier. The 2,4-dinitrophenylhydrazine (DNPH) sampling cartridges were supplied by the Waters Corporation (Milford, MA, USA). Petroleum ether, trichloroacetic acid, diethyl ether, disodium ethylenediaminetetraacetate (Na_2_EDTA), 95% ethanol, hydrochloric acid (≥36%), sodium hydroxide, sodium chloride, and chromatographic-grade acetonitrile were purchased from Sinopharm Chemical Reagent Co., Ltd. (Shanghai, China). n-Heptane, methanol, thiobarbituric acid, pyrogallic acid, synthetic zeolite, 2,4,6-trimethylpyridine, and sulfosalicylic acid (purity ≥98%) were obtained from Shanghai Aladdin Biochemical Technology Co., Ltd. (Shanghai, China).

### 2.2 Sample preparation and processing conditions

The pork belly was cut into strips measuring 0.2 × 0.2 × 6 cm. To reproduce traditional Chinese stir-frying under standardized conditions while minimizing operator-dependent variability, the samples were cooked using a Chushen H22 intelligent stir-frying machine (Lu'an Suoyi Electric Appliance Manufacturing Co., Ltd., Lu'an, Anhui, China). The appliance is equipped with an integrated overhead forced exhaust system featuring a centrifugal fan that operates at a constant exhaust airflow rate of 180 m^3^/h. The rectangular exhaust outlet (160 mm × 100 mm) incorporates a removable two-stage filtration unit, comprising a 40-mesh stainless steel screen (wire diameter: 0.15 mm) and a labyrinth-type baffle plate; the filtration unit has an effective open area ratio of 38%.

Brine was prepared by dissolving 450 g of sodium chloride and 50 g of monosodium glutamate in 1380 g of deionized water with continuous stirring until the solutes were completely dissolved. A soy sauce mixture was prepared by combining dark and light soy sauces at a volume ratio of 1:3 (*v*/v). Each treatment comprised 150 g of pork belly strips, 5 mL of peanut oil, 5 mL of cooking wine, 5 mL of the prepared soy sauce mixture, and 5 mL of brine. The processing conditions are summarized in Table 1. The stir-frying procedure comprised an initial heating phase (0–190 s), followed by a residual heat phase (190–360 s). To evaluate the effects of different COF environments on the aroma and flavor profiles of stir-fried pork belly, the fume control module was independently activated or deactivated during each phase. Four fume control regimes were established. In the OO treatment, the module remained activated during both the heating and residual heat phases (open-open). In the CC treatment, the module remained deactivated throughout both phases (closed-closed). In the CO treatment, the module was deactivated during the heating phase and activated during the residual heat phase (closed-open). Conversely, in the OC treatment, the module was activated during the heating phase and deactivated during the residual heat phase (open-closed). After completion of the residual heat phase, samples from each treatment were immediately frozen and stored at −20 °C until further analysis. The stir-frying experiment was conducted at a constant chamber temperature of 200 °C and a stirring speed of 25 r/min. As the cross-sectional area of the meat samples was small, their core temperature quickly reached equilibrium with the ambient temperature during the initial heating phase; therefore, the actual processing temperature of the meat samples was approximately 200 °C. The OO treatment, with the exhaust system continuously activated throughout both cooking stages, was employed as the baseline control group to simulate standard operating conditions of sustained exhaust ventilation during practical cooking operations. This configuration ensured the minimal accumulation of COFs and most closely approximated conventional culinary practice. In contrast, the CC, CO, and OC treatments were established as experimental groups, representing stage-specific exhaust interruption or delayed activation strategies. These experimental conditions were designed to systematically dissect the contribution of fume retention to the flavor profile of stir-fried pork belly and to characterize the stage-dependent effects of fume retention.

**Table 1:** Parameter settings of the intelligent stir-frying machine.

Time/s	0	8	10	40	70	155	190
Parameter setting	Set rotation speed	Set temperature	Add peanut oil	Add pork belly	Add cooking wine and soy sauce	Add brine	End

### 2.3 Collection and analysis of aldehydes and ketones in COFs

COFs generated during pork belly stir-frying were collected from the chamber of an intelligent stir-frying machine. A dual-channel constant-flow air sampler was operated at a flow rate of 500 mL/min (Wu, Peng, et al., 2021). The sampled air was drawn sequentially through a polytetrafluoroethylene (PTFE) filter, a potassium iodide (KI) ozone scrubber, and a 2,4-dinitrophenylhydrazine (DNPH) silica gel sampling cartridge. The PTFE filter removed particulate matter, whereas the KI scrubber minimized ozone interference with the DNPH derivatization reaction. Aldehydes and ketones were subsequently captured and derivatized within the DNPH cartridge. Each sample was collected for 30 min. After sampling, the cartridges were sealed in aluminum foil bags and stored at 4 °C in the dark until extraction. Each cartridge was eluted with acetonitrile into a 10 mL volumetric flask, and the eluate was made up to volume with the same solvent.

In accordance with the HJ 683–2014 standard method, 13 aldehydes and ketones were selected as target carbonyl compounds (De Araújo et al., 2022). Chromatographic separation and quantification were performed using a high-performance liquid chromatography (HPLC) system equipped with a Symmetry C18 column (4.6 × 250 mm, 5 μm) and a UV detector set at 360 nm. The injection volume was 20 μL, and an acetonitrile-water gradient was used as the mobile phase. Individual compounds were identified by comparing their retention times with those of the corresponding standards, and quantification was performed based on chromatographic peak areas.

### 2.4 Determination of thiobarbituric acid-reactive substances (TBARs)

TBARs were quantified as described by Wang et al. (2022), with minor modifications. A 2.5-g portion of minced and homogenized pork belly was combined with 25 mL of trichloroacetic acid solution and incubated with continuous agitation at 50 °C for 30 min. After cooling to room temperature, the suspension was filtered through two layers of filter paper. A 5 mL aliquot of the filtrate was subsequently combined with an equal volume of 0.02 mol/L thiobarbituric acid (TBA) solution. The reaction mixture was heated in a water bath at 90 °C for 30 min, and then cooled to room temperature. The absorbance was recorded at 532 nm. The TBARs concentrations were calculated from a calibration curve prepared using malondialdehyde (MDA) standard solutions and expressed as milligrams of MDA per kilogram of meat (mg MDA/kg).

### 2.5 Determination of fatty acid composition

Fatty acids were extracted and quantified according to the method described by Xie et al. (2022) with minor modifications. A 4.00 g portion of minced pork belly was accurately weighed and placed in a round-bottom flask. Subsequently, 2 mL of tridecanoin internal standard solution, approximately 100 mg of pyrogallic acid, and several boiling chips were added. The sample was then combined with 2 mL of 95% (*v*/v) ethanol and 4 mL of distilled water and homogenized thoroughly. The mixture was then subjected to acid hydrolysis to release the lipids. After hydrolysis, the flask was cooled to room temperature, and 10 mL of 95% (*v*/v) ethanol was added and mixed thoroughly. The hydrolysate was then transferred to a separatory funnel. The round-bottom flask and stopper were rinsed with 50 mL of a diethyl ether-petroleum ether mixture (1:1, *v*/v), and the rinsate was combined with the hydrolysate. The funnel was securely stoppered, shaken for 5 min, and allowed to stand for 10 min to facilitate the phase separation. The upper ether phase was collected in a 250-mL round-bottom flask. The extraction procedure was repeated thrice, and the organic phases were pooled. After the final extraction, the separatory funnel was rinsed with a diethyl ether-petroleum ether mixture, and the rinsate was combined with the pooled extracts. The combined organic extract was concentrated to near dryness under reduced pressure using a rotary evaporator to yield the lipid extract.

The lipid extract was saponified with 8 mL of 2% (*w*/*v*) sodium hydroxide in methanol. The flask was equipped with a reflux condenser, and the mixture was refluxed in a water bath at 80 °C until complete saponification was indicated by the disappearance of visible oil droplets. Subsequently, 7 mL of 15% (*w*/*v*) boron trifluoride in methanol was added, and the mixture was further refluxed at 80 °C for 2 min to afford fatty acid methyl esters (FAMEs). Upon completion of derivatization, 20 mL of n-heptane was added, and the mixture was shaken vigorously for 2 min. Saturated sodium chloride solution was then added, and the mixture was allowed to stand until complete phase separation was achieved. A 5-mL aliquot of the upper organic layer was transferred to a vial containing 4 g of anhydrous sodium sulfate, gently shaken for 1 min, and left to stand for 5 min to remove residual moisture. The dehydrated supernatant was subsequently transferred to a GC vial for chromatographic analysis.

FAMEs were subjected to gas chromatography-flame ionization detection (GC-FID) analysis on a TG-FAME column. The injector and detector were maintained at 280 °C. High-purity nitrogen was used as the carrier gas at 0.5 mL/min (split ratio, 1:30). The temperature program was optimized as follows: 80 °C (1 min), 20 °C/min to 160 °C (1.5 min), 3 °C/min to 205 °C (4 min), and 3 °C/min to 250 °C (2 min). Peak identification was achieved by matching retention times against a 37-component FAME standard. For quantification, tridecanoin was used as an internal standard, and fatty acid concentrations were determined from peak area ratios and reported as mg/100 g of sample.

### 2.6 Analysis of free amino acids (FAAs)

FAAs were extracted and quantified according to the method described by Jiang et al. (2024), with minor modifications. A 0.10-g portion of the freeze-dried sample was accurately weighed into a centrifuge tube and combined with 4 mL of 4% (m/v) sulfosalicylic acid solution. The suspension was vortex-mixed until homogeneous and sonicated at room temperature for 30 min, with the tube inverted at 5-min intervals. After sonication, the extract was allowed to stand for 10 min. A 1.5-mL aliquot of the supernatant was passed through a 0.22-μm hydrophilic membrane filter. The resulting filtrate was analyzed using an LA8080 automated amino acid analyzer.

### 2.7 Electronic-nose (E-nose) analysis

E-nose analysis was conducted according to the method described by Shen, Cai, et al. (2024), with minor modifications. A 3.00-g portion of minced and thoroughly homogenized pork belly was accurately weighed and placed in a headspace vial. The vial was immediately sealed and equilibrated at room temperature for 1 h. The headspace responses were measured using an E-nose system, with both the chamber and sample injection flow rates maintained at 200 mL/min. Signal acquisition was performed for 120 s per measurement, and each sample was analyzed in triplicates. The sensor array and corresponding nominal target analyte classes are summarized in Table 2.

**Table 2:** Correspondence of electronic nose sensors and detection substances.

No.	Sensor	Detection Substance
1	W1C	Aromatic compounds
2	W5S	Nitrogen oxides
3	W3C	Ammonia, aromatic molecules
4	W6S	Hydrogen compounds
5	W5C	Short-chain alkanes and aromatic components
6	W1S	Alkanes
7	W1W	Inorganic sulfides
8	W2S	Alcohols, aldehydes, ketones
9	W2W	Aromatic compounds, organic sulfur compounds
10	W3S	Alkanes and aliphatic compounds

### 2.8 Electronic-tongue (E-tongue) analysis

E-tongue analysis was performed according to the method described by Cheng et al. (2024), with minor modifications. A 4.00 g portion of pork belly was accurately weighed into a centrifuge tube and combined with 40 mL of ultrapure water. The mixture was homogenized at 10,000 rpm for 30 s and centrifuged at 10,000 rpm for 30 min. The resulting supernatant was filtered through filter paper and adjusted to a final volume of 80 mL using ultrapure water. The prepared extract was analyzed using the TS-5000Z E-tongue system. The taste responses evaluated were saltiness, richness, umami, aftertaste-A, aftertaste-B, astringency, bitterness, and sourness.

### 2.9 Headspace solid-phase microextraction coupled with gas chromatography–mass spectrometry (HS-SPME-GC–MS)

Volatile compounds were extracted and analyzed using HS-SPME-GC–MS, following Shen, Wang, et al. (2024). A 2 g portion of thoroughly homogenized pork belly was transferred to a 20 mL headspace vial, followed by the addition of 10 μL of 2,4,6-trimethylpyridine internal standard solution (6.3 μg/mL). The vial was immediately sealed and equilibrated in a water bath at 55 °C. A carboxen/polydimethylsiloxane (CAR/PDMS) fiber was exposed to the vial headspace approximately 1 cm above the surface of the sample. The volatile compounds were extracted at 55 °C for 40 min. After extraction, the fiber was inserted into the GC–MS injector and thermally desorbed at 230 °C for 5 min.

Chromatographic separation was performed using a DB-WAX capillary column (30 m × 0.25 mm i.d., 0.25 μm film thickness). High-purity helium was used as the carrier gas at a constant flow rate of 1.0 mL/min, and the samples were introduced in splitless mode. The oven temperature was initially maintained at 40 °C for 3 min and then increased to 200 °C at 5 °C/min. The temperature was then increased to 250 °C at 8 °C/min and maintained for 5 min. The mass spectrometer was operated in electron ionization mode at an ionization energy of 70 eV. The ion source and transfer line temperatures were maintained at 230 °C. Mass spectra were acquired in the full-scan mode over an *m*/*z* range of 33–500.

### 2.10 Gas chromatography–ion mobility spectrometry (GC-IMS) analysis

Volatile compound fingerprinting was performed using GC-IMS according to the method described by Yang et al. (2024), with minor modifications. A 2.00 g portion of pork belly was accurately weighed and placed in a 20 mL headspace vial. The vial was immediately sealed and incubated at 80 °C for 15 min. After incubation, a 500 μL aliquot of the headspace gas was automatically withdrawn and injected using an autosampler syringe maintained at 85 °C. High-purity nitrogen (≥ 99.99%) was used as both the carrier and drift gases. The initial carrier gas flow rate was maintained at 2 mL/min for 2 min and then increased linearly to 10 mL/min over 5 min. The flow rate was subsequently increased linearly to 100 mL/min over 25 min and maintained at this rate for 5 min. Each sample was analyzed in triplicate, and the measurements were averaged. Volatile compounds were tentatively identified by comparing their retention indices and drift times with reference values in the NIST and G.A.S. IMS databases.

### 2.11 Multivariate statistical analysis and data processing

All experiments were conducted using at least three independent replicates, and the results were presented as mean ± standard deviation (SD). Differences among the treatment groups were evaluated using one-way analysis of variance (ANOVA) with SPSS Statistics version 27.0 (IBM Corp., Armonk, NY, USA). Differences were considered statistically significant at *P* < 0.05. Principal component analysis (PCA) and orthogonal partial least-squares discriminant analysis (OPLS-DA) were performed using SIMCA-P version 14.1 (Umetrics, Umeå, Sweden). Data visualization and figure preparation were performed using Origin 2021 (OriginLab Corp., Northampton, MA, USA).

## 3 Results and discussion

### 3.1 HS-SPME-GC–MS analysis of pork belly volatiles

#### 3.1.1 Identification and quantification of volatile compounds

The volatile compound profiles of stir-fried pork belly subjected to four distinct fume-exhaust configurations were characterized by HS-SPME-GC–MS. A total of 62 volatile compounds were identified. As illustrated in the heatmap (Fig. 1A) and summarized in Table S1, normalized relative abundances were visualized using a color gradient, wherein red and cyan denote higher and lower relative abundances, respectively. The detected compounds included aldehydes (16), esters (2), alcohols (9), ketones (5), hydrocarbons (8), pyrazines (8), furans (6), and other compounds (8). Venn diagram analysis identified 49 nonredundant volatile compounds across the OO, OC, CO, and CC groups (Fig. 1B). Of these, 20 compounds were detected in all four groups, representing 40.8% of the 49 nonredundant compounds and indicating the presence of a common core volatile profile. The CC group contained the largest number of compounds (39) and the greatest number of treatment-specific compounds (6), demonstrating greater compositional richness and distinctiveness. The OO and CO groups each contained 33 compounds; however, only one compound was unique to the CO group, indicating limited treatment-specific differences. Overall, the four treatments shared the same constituents, with the CC group exhibiting the greatest diversity and specificity of volatile compounds.

**Fig. 1:**
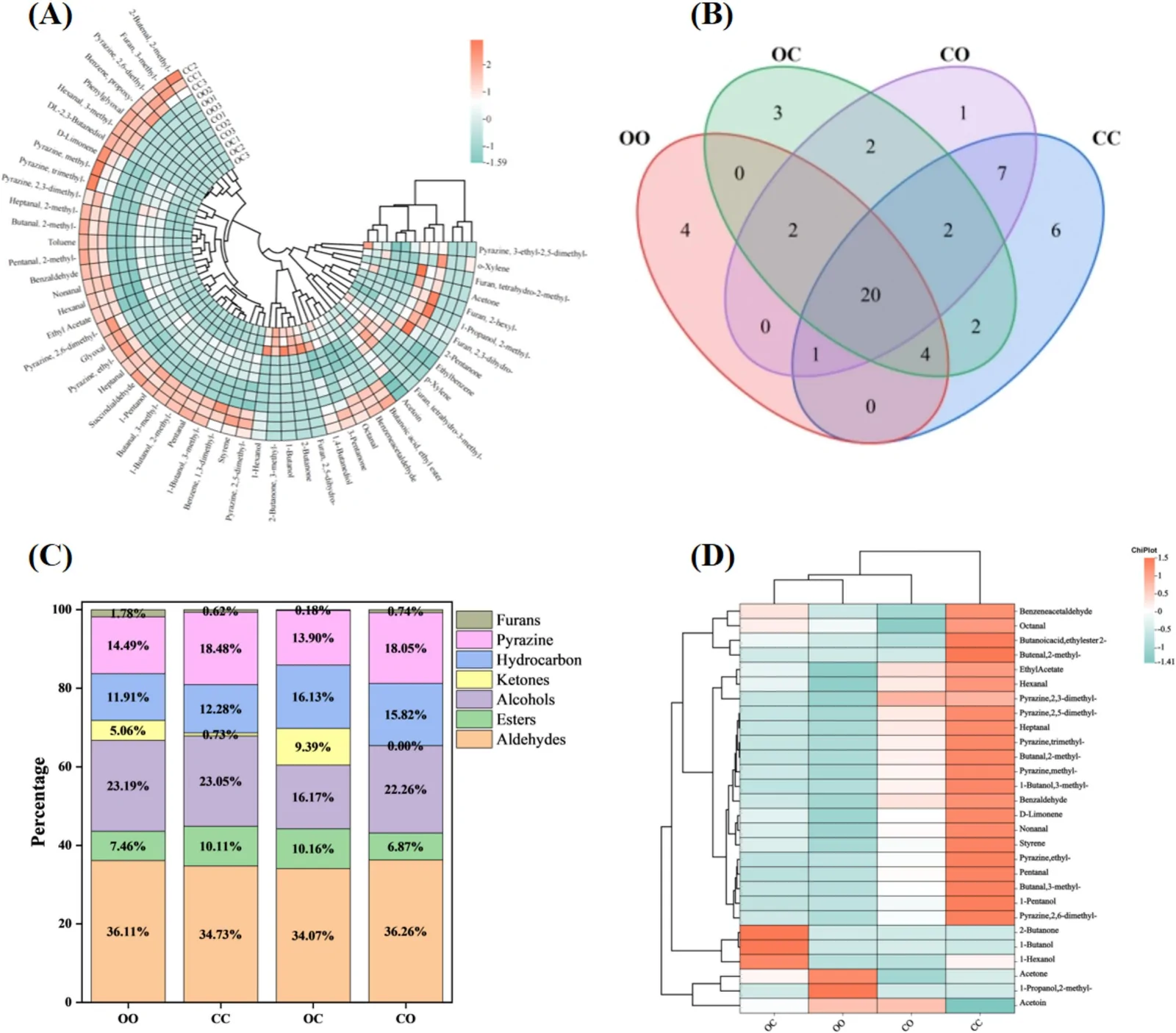
Comprehensive analysis of volatile flavor compounds in stir-fried pork belly under different oil fume exhaust modes. (A) Hierarchical clustering heatmap visualizing the relative abundance distribution of volatile compounds across treatment groups; (B) Venn diagram illustrating the shared and unique volatile components among the four sample groups; (C) Relative percentage composition of various volatile compound classes; (D) Heatmap analysis of key flavor contributors based on OAV.

Aldehydes are major contributors to the characteristic aromas generated during the thermal processing of meat products (Duan et al., 2024; Shen, Cai, et al., 2024). A total of 16 aldehydes were identified across the four treatments. Straight-chain saturated aldehydes, including nonanal, octanal, heptanal, and hexanal, were present at comparatively high concentrations. The concentrations of these aldehydes were significantly higher in the CC group than in the OO, OC, and CO groups (*P* < 0.05). Nonanal and octanal are primarily generated through the oxidative degradation of oleic acid, whereas hexanal predominantly originates from the oxidative cleavage of linoleic acid (Zhu, Niu, & Xiao, 2021). This distribution was consistent with the higher oleic and linoleic acid levels in the CC group, which may have provided a larger precursor pool for the formation of the corresponding aldehydes.

Alcohols can be generated through several biochemical and chemical pathways, including glucose metabolism, lipid oxidation, amino acid decarboxylation, and dehydrogenation. Nine alcohols were identified across the four treatments, with the CC group exhibiting the highest total alcohol concentration and the greatest number of individual alcohols. Pentanol, associated with fruity and aromatic notes, was detected exclusively in the CC and CO groups. Its concentration was significantly higher in the CC group than in the CO group (*P* < 0.05). These findings indicate that closed-exhaust conditions are associated with greater alcohol accumulation and a more diverse alcohol profile, potentially increasing the complexity of the overall aroma. Ketones are primarily generated through unsaturated fatty acid oxidation and amino acid degradation and are generally associated with fruity and floral aromas (Lorenzo & Domínguez, 2014). Although ketones typically have relatively high odor thresholds, they can contribute to the overall aroma through interactions with aldehydes and other volatile compounds. Esters are predominantly formed through esterification reactions between organic acids and alcohols (Domínguez et al., 2019). Two esters, ethyl acetate and ethyl butyrate, were detected in pork belly samples.

The hydrocarbons comprised aliphatic hydrocarbons, aromatic hydrocarbons, and terpenes. Aliphatic hydrocarbons are commonly generated through lipid oxidation, whereas aromatic hydrocarbons may originate from the oxidative degradation of their precursors (Zhu et al., 2023). The principal aromatic hydrocarbons detected in stir-fried pork belly were toluene, o-xylene, 1,3-dimethylbenzene, and styrene. Because hydrocarbons generally have relatively high odor thresholds, their direct contribution to the overall aroma of pork belly is likely to be limited. d-limonene, a naturally occurring monoterpene associated with a characteristic citrus aroma, may contribute to the distinctive aromatic profile of cooked meat (Feng et al., 2017). Pyrazines and furans are important heterocyclic compounds generated during the thermal processing of meat. These compounds can arise through Maillard-associated pathways, involving Strecker degradation and aldehyde condensation reactions (Zhou et al., 2022). Eight pyrazines and six furans were identified in the four treatments. The summed concentrations of both compound classes were significantly higher in the CC group than in the OO, OC, and CO groups (*P* < 0.05). Therefore, the closed-exhaust condition was associated with enhanced accumulation of pyrazines and furans, which may have strengthened the characteristic cooked-meat aroma of the pork belly.

The relative distribution of the seven volatile compound classes differed markedly among the four treatments (Fig. 1C). The CC group exhibited the highest relative abundance of pyrazines (18.48%), indicating a predominantly Maillard-associated volatile profile. In contrast, ketones and furans represented only 0.73% and 0.62% of the CC profile, respectively. Although the absolute furan concentration was higher in the CC group, its low proportional contribution illustrates the distinction between the absolute concentration and relative composition. The OC group exhibited the highest relative abundances of ketones (9.39%) and hydrocarbons (16.13%), suggesting a comparatively greater contribution from lipid oxidation-derived volatiles. Alcohols, furans, and pyrazines accounted for 16.17%, 0.18%, and 13.90%, respectively, whereas ketones and hydrocarbons were more prominent, producing a profile weighted toward lipid oxidation-derived volatiles. Both the CO and CC groups exhibited pyrazine-rich profiles, with pyrazines accounting for 18.05% and 18.48% of the volatile compounds, respectively. In this group, esters represented 6.87% of the profile, whereas ketones were not detected, potentially limiting the chemical complexity. In contrast, the OO group exhibited the most even distribution of compound classes. Aldehydes, alcohols, and pyrazines contributed 36.11%, 23.19%, and 14.49%, respectively, without a pronounced dominance or depletion of any major class.

#### 3.1.2 Odor activity values (OAVs)

OAVs were calculated to estimate the contribution of individual aroma-active compounds to the overall aroma profile of the pork belly samples. OAVs were defined as the ratio of the semiquantitative concentration of a volatile compound to its reported odor threshold in water (Yang et al., 2022). Odor-threshold values were obtained from the literature (Li et al., 2022; Zhu, Guo, et al., 2021). Compounds with OAVs ≥1 are generally regarded as aroma-active, whereas higher OAVs indicate a greater potential contribution to the overall aroma (Zhu, Niu, & Xiao, 2021). As shown in Fig. 1D, 3-methylbutanal, 2-methylbutanal, hexanal, ethyl acetate, ethyl butyrate, and 2,5-dimethylpyrazine exhibited OAVs ≥1 and were identified as important aroma-active compounds in stir-fried pork belly. All six compounds exhibited higher OAVs in the CC group than in the other treatments, suggesting that the CC treatment produced the greatest potential aroma impact. The increased OAVs, in conjunction with the greater volatile diversity in the CC group, suggest that this treatment yielded a more intense and compositionally complex aroma profile. Such a pattern may be attributable to differential formation and retention of volatile compounds, including lipid oxidation- and hydrolysis-derived products, under the closed-exhaust regime.

### 3.2 GC-IMS analysis

GC**-**IMS was used to generate characteristic fingerprints of volatile organic compounds (VOCs) in the pork belly samples (Shen, Wang, et al., 2024). Fig. 2A presents the GC-IMS two-dimensional topographic plots revealing the distribution profiles of VOCs in stir-fried pork belly under different exhaust ventilation regimes. The ordinate denotes the GC retention time, and the abscissa represents the ion mobility drift time. Pronounced differences were observed in both the spatial distribution patterns and relative signal intensities among the groups. The detailed compound assignments are provided in Table S2. The differential plots revealed marked treatment-dependent variations in the VOCs profiles (Fig. 2B). To facilitate visual comparison, the OO group was designated as the reference group, and differential spectra were generated for the OC, CC, and CO groups. The white regions indicate no detectable difference between the treatment and OO reference, whereas the red and blue regions represent higher and lower signal intensities, respectively. Red regions predominated in the differential spectra of the OC, CO, and CC groups, indicating that numerous VOCs were present at higher relative abundances than in the OO group. The fingerprint plot further demonstrated substantial differences in the volatile compositions among the four treatments (Fig. 2C). Each column represents an individual volatile feature, and the color intensity reflects its relative signal abundance. These variations indicate that the fume exhaust regime markedly affected the volatile profile of the stir-fried pork belly. GC-IMS resolved 70 volatile features across the four treatments, of which 65 were tentatively identified and five were unidentified. The identified compounds comprised 16 aldehydes, 13 alcohols, 8 ketones, 19 esters, 2 acids, 2 furans, 2 pyrazines, and 3 alkenes. Although esters constituted the most numerous chemical class, aldehydes exhibited the greatest overall abundance. Aldehydes are predominantly generated through the oxidative degradation of fatty acids. Because these compounds generally have low odor thresholds, they may contribute substantially to the aroma of pork belly, even at relatively low concentrations. Alcohols were also prominent across all treatments and included 1-hexanol, 1-pentanol, 2-ethoxyethanol, 3-methyl-1-butanol, 1-butanol, 2-methyl-1-propanol, 1-propanol, and ethanol. The major ketones were 3-hydroxy-2-butanone, 2-butanone, and acetone. The summed relative abundance of ketones was significantly lower in the OO group than in the other three groups. Esters impart fruity notes and contribute to the overall aromatic complexity of meat products. The principal esters were ethyl acetate, propyl succinate, and butyl acetate. The CC group exhibited a significantly higher relative ester abundance than the OO, OC, and CO groups. Organic acids can originate from lipid oxidation and microbial amino acid metabolism. However, their relative abundance remained low across all treatments. Pyrazines and furans exhibited low signal abundances in the GC-IMS profiles.

**Fig. 2:**
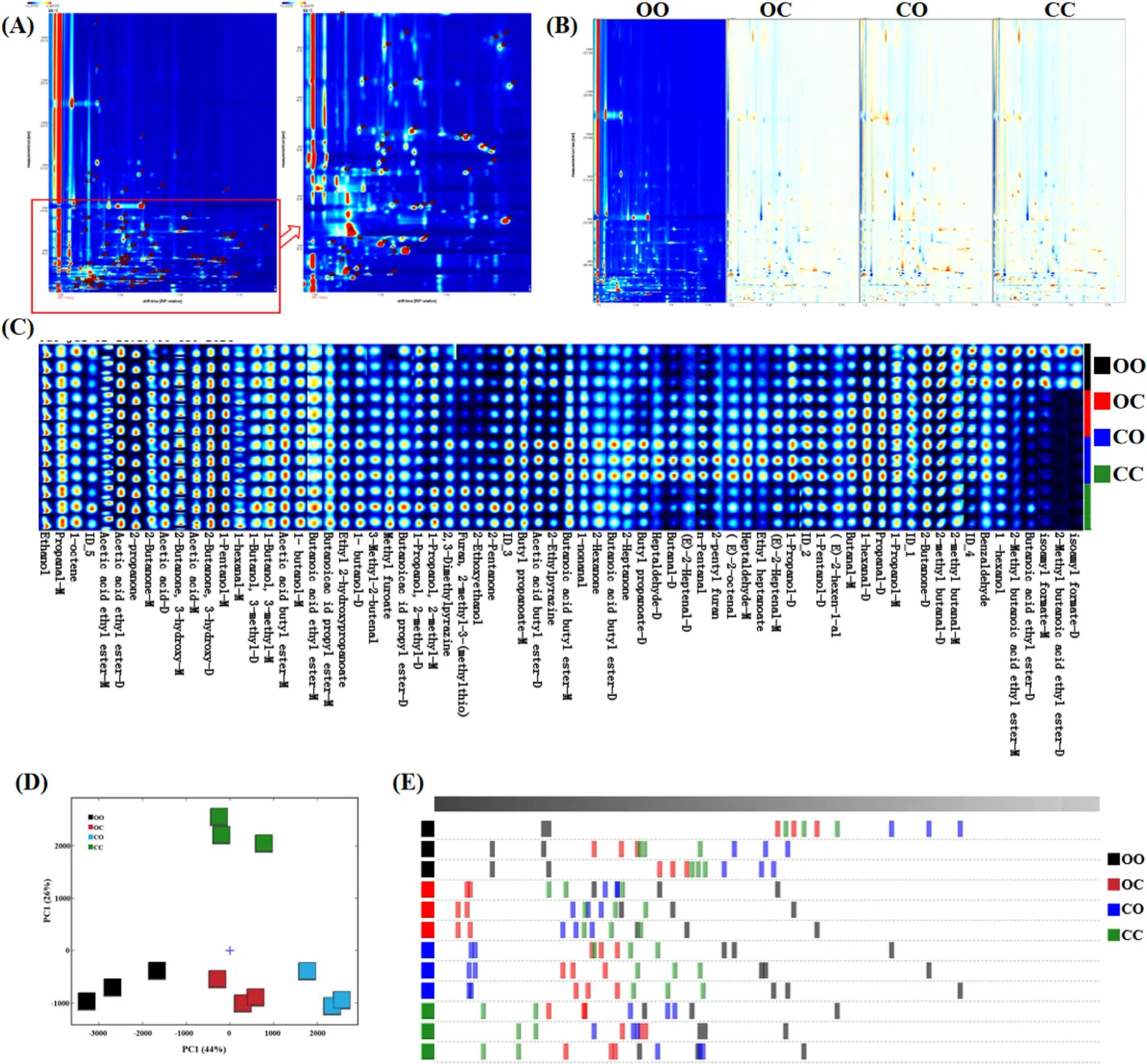
GC-IMS analysis of volatile organic compounds in stir-fried pork belly under different oil fume exhaust modes. (A) Qualitative analysis of volatile organic compounds; (B) Two-dimensional GC-IMS topographic plots; (C) Gallery Plot fingerprint spectra (differential visualization with OO group as reference); (D) Principal component analysis (PCA); (E) Nearest-neighbor similarity map among samples.

Principal component analysis (PCA) provided an overview of the treatment-dependent variations in volatile profiles (Fig. 2D). The first two principal components collectively explained 70% of the total variance, indicating that the two-dimensional score plot adequately represented the primary differences between the samples. The CC samples were clearly separated from the OO, OC, and CO samples, demonstrating that the CC treatment produced a distinct volatile profile. In contrast, the OC and CO samples were positioned close to each other, indicating a comparatively high degree of compositional similarity. A nearest-neighbor analysis based on the Euclidean distance was subsequently used to complement the PCA results. With the OO group as the reference, the distances of the treatment groups increased progressively from OC to CO to CC (Fig. 2E). Accordingly, the OO and OC groups exhibited the greatest similarity, whereas the CC group exhibited the greatest dissimilarity. This pattern was consistent with the distribution of PCA scores. Collectively, these results demonstrate that the fume-exhaust configuration markedly altered the volatile composition of stir-fried pork belly, with the closed-exhaust CC condition producing the greatest deviation from the OO reference.

### 3.3 E-nose and E-tongue analyses

E-nose was used to distinguish treatment-dependent differences in the volatile fingerprints of pork belly samples (Hui et al., 2025). As shown in Fig. 3A, the response patterns of the 10 E-nose sensors varied markedly among the samples subjected to the four fume-exhaust configurations. The W1W sensor (nominally responsive to sulfides), W2W (organic sulfides), W1S (methyl-containing compounds), and W2S (alcohols such as ethanol) exhibited stronger signals than the other sensors (Hui et al., 2023). These response patterns suggest that the sample headspaces contained appreciable quantities of compounds to which these sensors are broadly responsive, including alcohols and sulfur-containing volatiles. The CC group produced the strongest W1W, W2W, and W2S responses, indicating that its volatile fingerprint differed substantially from those of the other three groups. Because E-nose sensors are cross-sensitive, these responses reflect collective changes in multiple VOCs rather than the concentrations of individual compounds. Nevertheless, the overall response pattern was broadly consistent with the treatment-related differences observed by GC–MS and GC-IMS. PCA was subsequently performed using E-nose response data (Fig. 3B). PC1 and PC2 explained 90.72% and 9.01% of the total variance, respectively, providing a cumulative explained variance of 99.73%. Thus, the two-dimensional score plot retained nearly all of the variations in the E-nose dataset and adequately represented the treatment-dependent differences in the volatile fingerprints. The relatively tight clustering of the replicate measurements suggests satisfactory analytical repeatability.

**Fig. 3:**
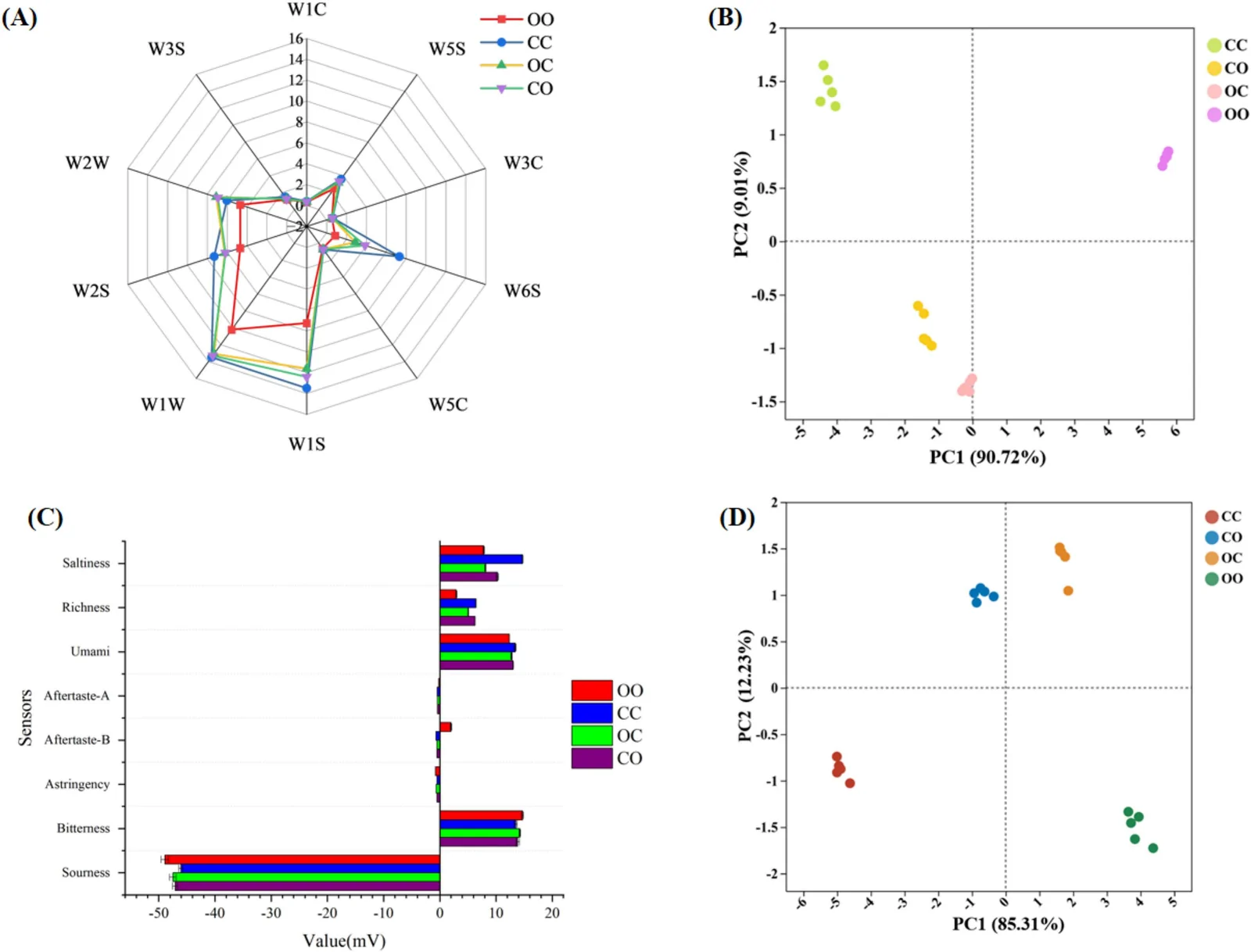
(A) Electronic nose sensor response; (B) Electronic nose PCA score plot; (C) Electronic tongue sensor response; (D) Electronic tongue PCA score plot.

The E-tongue response profiles are shown in Fig. 3C. The four treatments produced discernible responses for sourness, bitterness, umami, and saltiness, among the eight taste attributes evaluated. The CC group exhibited the strongest saltiness response, followed by the CO, OC, and OO groups. The same treatment ranking was observed for umami. Conversely, the CC group exhibited weaker bitterness and sourness responses than the other three groups. PCA of the E-tongue data further distinguished the instrumental taste profiles of the four treatments (Fig. 3D). PC1 and PC2 explained 85.31% and 12.23% of the total variance, respectively, collectively accounting for 97.54%. Because most of the variation was captured by PC1, the treatment-dependent differences were predominantly expressed along this axis. The CC and OO groups were positioned farthest apart, indicating the most distinct instrumental taste profiles, consistent with their contrasting saltiness and umami responses.

### 3.4 FAAs profiles and taste activity values

FAAs are important taste-active constituents and precursors of thermally generated aroma compounds in meat. They contribute directly to the fundamental taste attributes, including sweetness, bitterness, umami, and saltiness. FAAs also participate in Strecker degradation and Maillard reactions during thermal processing, generating numerous volatile aroma compounds (Wang et al., 2024). In this study, 17 free amino acids were identified and quantified in pork belly samples, and their concentrations are listed in Table 3. The CC group had the highest total free amino acid (TFAA) concentration at 760.99 mg/100 g, which was 27.3% higher (*P* < 0.05) than that of the OO group (597.99 mg/100 g). The TFAA concentrations in the CO and OC groups were 741.80 mg/100 g and 722.42 mg/100 g, respectively. The substantial elevation in TFAA concentration observed in the CC group can be reasonably ascribed to the high-temperature, high-humidity microenvironment generated within the closed cooking chamber, which likely accelerated protein thermal degradation and consequently promoted the liberation of FAAs. With respect to taste-active amino acids, the CC and CO groups were characterized by relatively high concentrations of glutamic acid (Glu) and alanine (Ala), which may constitute a critical source of the savory taste in thermally processed pork belly (Hwang et al., 2019). Additionally, the contents of the sweet-taste amino acids glycine (Gly), serine (Ser), and threonine (Thr) in the OO group were significantly lower than those in the other three groups (*P* < 0.05), whereas no significant differences were observed among the CC, OC, and CO groups (*P* > 0.05). Apart from their direct contribution to sweetness, Gly, Ser, and Thr can undergo Strecker degradation to generate corresponding aldehydes, thereby facilitating the formation of pyrazine-derived flavor compounds (Zhang et al., 2020). This finding is consistent with the aforementioned observation that the OO group exhibited a relatively low proportion of pyrazine compounds in the flavor analysis, lending further support to this association.

**Table 3:** Effects of different cooking emission patterns on the FAAs and TAVs of stir-fried pork belly.

Taste compounds	Concentrations (mg/100 g)				Taste threshold (mg/100 g)^A^	TAVs			
	OO	CC	OC	CO		OO	CC	OC	CO
Sweet(+)									
Ala	80.49 ± 1.66^c^	104.51 ± 2.21^b^	114.05 ± 2.81^a^	110.36 ± 7.33^ab^	60	1.34 ± 0.03^c^	1.74 ± 0.04^b^	1.90 ± 0.05^a^	1.84 ± 0.12^ab^
Ser	20.95 ± 0.24^b^	27.52 ± 0.73^a^	26.26 ± 0.65^a^	26.97 ± 1.77^ab^	150	0.14 ± 0.00^b^	0.18 ± 0.00^a^	0.18 ± 0.00^a^	0.18 ± 0.01^ab^
Thr	17.6 ± 0.04^b^	22.24 ± 0.59^a^	21.55 ± 0.54^a^	22.09 ± 1.36^a^	260	0.07 ± 0.00^b^	0.09 ± 0.00^a^	0.08 ± 0.00^a^	0.08 ± 0.01^a^
Gly	29.11 ± 0.54^b^	37.02 ± 0.79^a^	37.85 ± 0.96^a^	36.86 ± 2.12^a^	130	0.22 ± 0.00^b^	0.28 ± 0.01^a^	0.29 ± 0.01^a^	0.28 ± 0.02^a^

Bitter/sweet (+)									
Arg	7.97 ± 0.11^d^	11.69 ± 0.26^b^	10.11 ± 0.41^c^	10.89 ± 0.59^a^	50	0.16 ± 0.00^d^	0.23 ± 0.01^b^	0.20 ± 0.01^c^	0.22 ± 0.01^a^
Pro	20.88 ± 0.74^b^	44.5 ± 3.89^a^	25.12 ± 1.53^b^	24.27 ± 2.20^b^	300	0.07 ± 0.00^b^	0.15 ± 0.01^a^	0.08 ± 0.01^b^	0.08 ± 0.01^b^
Val	19.72 ± 0.17^c^	25.9 ± 0.72^a^	21.83 ± 0.68^bc^	23.95 ± 1.92^ab^	40	0.49 ± 0.00^c^	0.65 ± 0.02^a^	0.55 ± 0.02^bc^	0.60 ± 0.05^ab^
Lys	19.34 ± 0.27^b^	26.08 ± 0.45^a^	24.66 ± 0.70^a^	25.67 ± 1.58^a^	50	0.39 ± 0.01^b^	0.52 ± 0.01^a^	0.49 ± 0.01^a^	0.51 ± 0.03^a^

Umami (+)									
Asp	25.02 ± 0.23^c^	30.71 ± 1.22^a^	31.82 ± 1.18^a^	27.73 ± 1.36^b^	100	0.25 ± 0.00^c^	0.31 ± 0.01^a^	0.32 ± 0.01^a^	0.28 ± 0.01^b^
Glu	260.46 ± 3.19^b^	318.91 ± 8.79^a^	303.84 ± 10.16^a^	316.83 ± 20.12^a^	30	8.68 ± 0.11^b^	10.63 ± 0.29^a^	10.13 ± 0.34^a^	10.56 ± 0.67^a^

Bitter (−)									
Tyr	9.94 ± 0.40^b^	12.89 ± 0.76^a^	10.23 ± 0.16^b^	12.34 ± 0.95^a^	90	0.11 ± 0.00^b^	0.14 ± 0.01^a^	0.11 ± 0.00^b^	0.14 ± 0.01^a^
Phe	13.01 ± 0.42^c^	17.88 ± 0.42^a^	14.71 ± 0.41^b^	16.46 ± 1.26^a^	90	0.14 ± 0.00^c^	0.20 ± 0.00^a^	0.16 ± 0.00^b^	0.18 ± 0.01^a^
His	7.53 ± 0.30^b^	10.61 ± 0.43^a^	9.74 ± 0.35^a^	10.31 ± 0.66^a^	20	0.38 ± 0.01^b^	0.53 ± 0.02^a^	0.49 ± 0.02^a^	0.52 ± 0.03^a^
Lle	19.12 ± 0.25^b^	20.78 ± 2.68^ab^	20.68 ± 0.17^ab^	22.69 ± 1.51^a^	90	0.21 ± 0.00^b^	0.23 ± 0.03^ab^	0.23 ± 0.00^ab^	0.25 ± 0.02^a^
Leu	25.69 ± 0.29^b^	31.59 ± 1.88^a^	27.67 ± 0.92^b^	31.77 ± 2.38^a^	190	0.14 ± 0.00^b^	0.17 ± 0.01^a^	0.15 ± 0.00^b^	0.17 ± 0.01^a^

Bitter/sweet /astringent (−)									
Cys	11.04 ± 0.03^a^	11.29 ± 0.59^a^	11.47 ± 1.34^a^	10.64 ± 0.57^ab^	48	0.23 ± 0.00^a^	0.24 ± 0.01^a^	0.24 ± 0.03^a^	0.22 ± 0.01^ab^
Met	10.11 ± 0.22^c^	6.85 ± 0.15^d^	10.83 ± 0.11^b^	11.94 ± 0.58^a^	30	0.34 ± 0.01^c^	0.23 ± 0.00^d^	0.36 ± 0.00^b^	0.40 ± 0.02^a^
TFAA	597.99 ± 8.28^b^	760.99 ± 19.11^a^	722.42 ± 18.67^a^	741.8 ± 42.17^a^					

Note: Experimental data are expressed as mean ± standard error. Different lowercase letters indicate significant differences between groups (*P* < 0.05). ^A^Taste threshold value (mg/ml) and taste of free amino acids in water (Chen & Zhang, 2007; Duppeti et al., 2022). Ala, alanine; Ser, serine; Thr, threonine; Gly, glycine; Arg, arginine; Pro, proline; Val, valine; Lys, lysine; Asp, aspartic acid; Glu, glutamic acid; Tyr, tyrosine; Phe, phenylalanine; His, histidine; Ile, isoleucine; Leu, leucine; Cys, cysteine; Met, methionine. TFAAs, total free amino acids; TAV, taste activity value.

Taste activity values (TAVs), calculated as the ratio of the measured concentration of a compound to its taste threshold in aqueous solution, reflect the contribution of individual tastants to the overall taste profile. Compounds with TAVs >1 are considered to be taste-active and are recognized as key contributors to flavor perception (Chen & Zhang, 2007; Shen, Cai, et al., 2024). Based on the quantitative data for free amino acids, the TAVs for each component were calculated to identify the key taste-active amino acids that influence flavor attributes. The results are shown in Table 3. Among the seventeen FAAs identified, only Ala and Glu exhibited TAVs greater than 1, indicating that these two amino acids are the most critical contributors to the taste profile of pork belly. Notably, the TAV of Glu substantially exceeded that of Ala, establishing its absolute predominance in umami contribution. The CC group displayed the highest Glu concentration, with a TAV of 10.63, thereby supporting the pronounced umami characteristics observed in this group. Furthermore, the Met concentration in the CC group was significantly lower than that in the OO group, with a reduction of 32.2% (*P* < 0.05). In conjunction with the elevated response values of the W1W and W2W sensors to sulfur-containing and aromatic compounds, as revealed by electronic nose analysis, it is hypothesized that Met may have been extensively converted into sulfur-containing volatile flavor compounds under CC treatment conditions. Concurrently, the CC group exhibited a markedly higher Pro concentration, reaching 2.13 times that of the OO group, suggesting that Pro may serve as an important precursor for the formation of pyrazine compounds.

### 3.5 Free fatty acid composition and lipid oxidation in pork belly

Free fatty acids (FFAs) primarily originate from the lipolysis of phospholipids and triacylglycerols (Wang et al., 2016), serving as important flavor precursors in meat products and as critical substrates for the generation of volatile flavor compounds, including aldehydes, alcohols, and ketones (Wu, Yang, et al., 2021). As presented in Table 4, a total of 24 fatty acids were identified and quantified in the pork belly samples, comprising 9 saturated fatty acids (SFAs), 7 monounsaturated fatty acids (MUFAs), and 8 polyunsaturated fatty acids (PUFAs). The different fume-exhaust configurations of the intelligent stir-frying machine during the cooking process exerted significant effects on the fatty acid composition and contents of pork belly (*P* < 0.05). Compared with the OO, OC, and CO treatment groups, the CC group exhibited significant reductions in both total FFA and SFA contents, accompanied by varying reductions in MUFA and PUFA contents.

**Table 4:** Effect of different cooking fume exposure conditions on fatty acid contents in pork belly (mg/100 g).

FFAs	OO	CC	OC	CO
C12:0	0.17 ± 0.01^a^	0.11 ± 0.00^c^	0.13 ± 0.00^b^	0.06 ± 0.00^d^
C14:0	2.20 ± 0.02^a^	1.14 ± 0.10^c^	1.88 ± 0.01^b^	0.64 ± 0.00^d^
C15:0	ND	ND	0.14 ± 0.11	0.17 ± 0.22
C16:0	25.94 ± 0.09^a^	11.52 ± 0.03^c^	21.16 ± 1.38^b^	22.4 ± 0.82^b^
C17:0	0.90 ± 0.01^a^	0.20 ± 0.00^c^	0.26 ± 0.00^b^	0.12 ± 0.00^d^
C18:0	27.26 ± 0.13^a^	11.62 ± 0.04^d^	19.10 ± 0.10^b^	12.41 ± 0.72^c^
C20:0	1.17 ± 0.01^a^	0.57 ± 0.04^c^	0.76 ± 0.01^b^	0.36 ± 0.01^d^
C22:0	2.01 ± 0.08^a^	0.88 ± 0.01^c^	1.12 ± 0.04^b^	0.60 ± 0.02^d^
C23:0	11.07 ± 0.52^a^	4.36 ± 1.88^b^	4.42 ± 0.13^b^	4.45 ± 0.17^b^
**SFAs**	70.72 ± 0.71^a^	30.40 ± 2.00^d^	48.97 ± 1.51^b^	41.21 ± 0.59^c^
C14:1	ND	ND	0.13 ± 0.05	0.66 ± 0.04
C16:1	4.80 ± 0.02^a^	2.10 ± 0.00^b^	ND	1.26 ± 0.00^c^
C17:1	0.53 ± 0.01^a^	0.29 ± 0.00^c^	0.34 ± 0.00^b^	0.23 ± 0.00^d^
C18:1n9c	53.55 ± 0.22^a^	20.70 ± 0.07^c^	40.41 ± 0.19^b^	19.66 ± 1.07^c^
C18:1n9t	0.25 ± 0.02^b^	0.10 ± 0.05^c^	0.09 ± 0.00^c^	7.04 ± 0.05^a^
C20:1	1.33 ± 0.01^a^	0.49 ± 0.00^c^	0.72 ± 0.01^b^	0.26 ± 0.00^d^
C22:1n9	ND	0.17 ± 0.04	0.10 ± 0.01	0.33 ± 0.03
**MUFAs**	60.46 ± 0.25^a^	23.85 ± 0.09^c^	41.79 ± 0.16^b^	29.44 ± 1.1^c^
C18:2n6t	1.41 ± 0.79^a^	0.40 ± 0.07^bc^	0.20 ± 0.01^cd^	1.05 ± 0.30^ab^
C18:2n6c	0.87 ± 0.01^a^	0.51 ± 0.05^c^	0.63 ± 0.00^b^	0.20 ± 0.00^d^
C18:3n6	2.08 ± 0.02^a^	0.95 ± 0.04^c^	1.52 ± 0.01^b^	0.55 ± 0.01^d^
C18:3n3	1.02 ± 0.00^a^	0.17 ± 0.00^c^	0.19 ± 0.00^b^	0.19 ± 0.00^b^
C20:2	8.25 ± 0.07^a^	1.29 ± 0.01^c^	0.99 ± 0.00^d^	1.69 ± 0.01^b^
C20:3n6	0.91 ± 0.10	ND	ND	ND
C22:2	ND	0.31 ± 0.02	ND	ND
C20:5n3	2.00 ± 0.17^a^	0.76 ± 0.05^b^	0.94 ± 0.07^b^	ND
**PUFAs**	16.54 ± 0.68^a^	4.38 ± 0.09^b^	4.46 ± 0.09^b^	3.67 ± 0.31^c^
**UFAs**	77.00 ± 0.70^a^	28.23 ± 0.16^c^	46.25 ± 0.23^b^	33.11 ± 1.33^c^
**TFFAs**	147.72 ± 1.12^a^	58.63 ± 2.14^d^	95.22 ± 1.59^b^	74.32 ± 1.73^c^

Note: Experimental data are expressed as mean ± standard error. Different lowercase letters indicate significant differences between groups (*P* < 0.05). ND indicates not detected in the sample. FFAs: free fatty acids; SFAs: saturated fatty acids; MUFA: monounsaturated fatty acid; PUFA: polyunsaturated fatty acid; MUFAs+PUFAs: total unsaturated fatty acids; TFFA: total free fatty acids.

These findings suggest that the CC treatment conditions were associated with a relatively higher degree of lipid oxidation. Given that the susceptibility of fatty acids to oxidation follows the order PUFA > MUFA > SFA, the concurrent reduction across all fatty acid classes in the CC group may reflect the combined effects of oxidative reactions and lipolytic processes. Among all treatment groups, oleic acid was the most abundant MUFA, and its content differed significantly among the groups (*P* < 0.05). The OO group exhibited the highest oleic acid content (53.55 mg/100 g), whereas the CC and CO groups had the lowest contents. This disparity may be attributed to the more extensive oxidation of oleic acid under CC and CO treatment conditions, with oxidative cleavage generating volatile flavor compounds such as heptanal, octanal, and nonanal (Ba et al., 2013). Furthermore, the linoleic acid content in the OO group was significantly higher than that in the other three groups (*P* < 0.05). This may be attributed to the continuously open exhaust in the OO treatment, which may have reduced the oxygen concentration and thermal accumulation within the system, thereby partially inhibiting the autoxidation of linoleic acid and diminishing its conversion into secondary oxidation products such as hexanal.

The extent of secondary lipid oxidation was assessed by measuring TBARS. As shown in Fig. 4, the TBARS values differed significantly among the fume-exhaust configurations (*P* < 0.05). The values decreased in the following order: CC (0.992 mg MDA/kg) > CO (0.645 mg MDA/kg) > OC (0.581 mg MDA/kg) > OO (0.363 mg MDA/kg). This pattern indicates that progressively greater exhaust closure was associated with more extensive lipid oxidation, with the CC condition producing the highest TBARS value. The higher TBARS value in the CO group than in the OC group further suggests that exhaust closure during the active heating phase was more strongly associated with lipid oxidation than closure during the residual heat phase. Lipids are major precursors of aroma compounds in thermally processed meat, and differences in their oxidative degradation can substantially modify the resulting aroma and overall flavor profiles (Zou et al., 2018). Collectively, the decreased concentrations of unsaturated fatty acids and elevated TBARS values under CC conditions suggest that the effect of the fume-exhaust mode on pork belly flavor was associated with the extent of lipid oxidation.

**Fig. 4:**
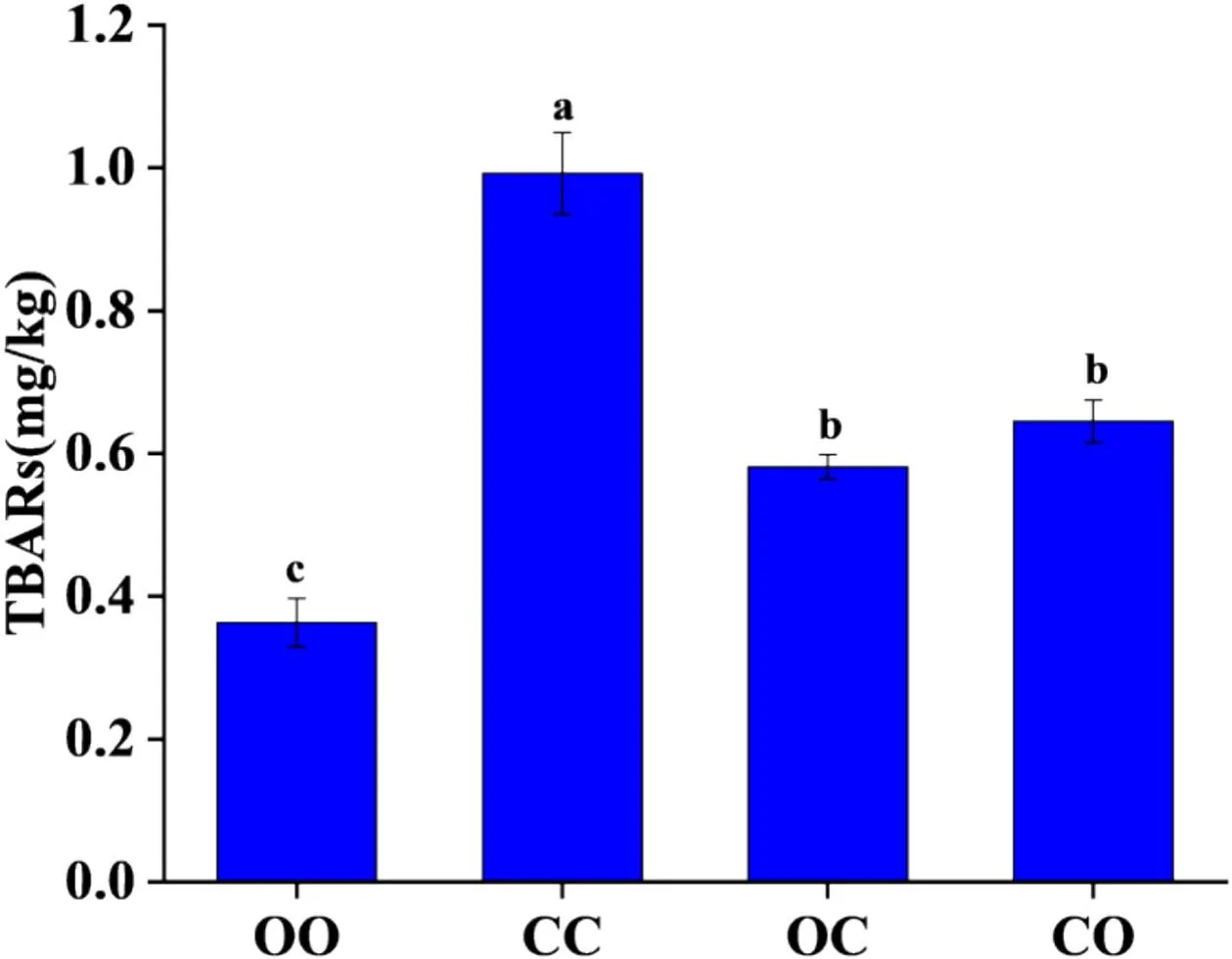
Effects of Different Cooking Fume Emission Patterns on Lipid Oxidation in Stir-Fried Pork Belly.

### 3.6 Aldehyde and ketone concentrations in cooking fumes during pork belly stir-frying

Under identical stir-frying conditions, the effects of different operating states of the fume-control module on aldehyde and ketone concentrations in the cooking environment were compared (Table 5). Overall, the fume control module exerted a significant regulatory effect on the concentrations of aldehydes and ketones in the cooking environment. The total aldehyde and ketone contents differed significantly among the four treatment groups (*P* < 0.05), with the OO, CC, OC, and CO groups exhibiting values of 750.75 μg/m^3^, 4868.89 μg/m^3^, 1886.19 μg/m^3^, and 4215.14 μg/m^3^, respectively. The total aldehyde and ketone content in the CC group was approximately 6.5 times that of the OO group, indicating that maintaining the fume control module in the closed state throughout the entire stir-frying process (i.e., with no fume exhaust) resulted in substantial accumulation of aldehydes and ketones in the cooking environment. Notably, the total aldehyde and ketone content in the OC group was significantly lower than that in the CO group (*P* < 0.05), suggesting that cooking fumes generated during the heating phase made a more pronounced contribution to the total aldehyde and ketone concentration in the environment.

**Table 5:** Environmental fume concentrations during the stir-frying of pork belly using different cooking methods.

Compound	OO	CC	OC	CO
Formaldehyde	122.55 ± 3.36^b^	248.17 ± 47.17^a^	232.04 ± 28.54^a^	196.99 ± 17.04^a^
Acetaldehyde	320.29 ± 7.37^d^	2978.89 ± 104.80^a^	1074.99 ± 68.90^c^	2578.47 ± 97.25^b^
Acrolein/Acetone	ND	25.89 ± 3.59	ND	36.39 ± 1.08
Propanal	ND	216.19 ± 12.72^a^	63.94 ± 3.99^c^	160.77 ± 3.58^b^
Crotonaldehyde	ND	ND	ND	ND
Methacrolein	ND	ND	ND	ND
2-Butanone	ND	ND	ND	ND
Butanal	24.96 ± 9.37^b^	734.76 ± 85.80^a^	104.85 ± 6.74^b^	694.00 ± 12.59^a^
Benzaldehyde	143.31 ± 11.43	41.08 ± 1.72	ND	ND
Pentanal	134.03 ± 7.84^a^	160.31 ± 33.76^a^	46.12 ± 3.78^c^	86.69 ± 1.00^b^
Methylbenzaldehyde	ND	ND	ND	ND
Hexanal	5.60 ± 0.30^b^	463.62 ± 93.98^a^	371.91 ± 9.84^a^	461.83 ± 39.87^a^
Total	750.75 ± 15.47^d^	4868.89 ± 380.72^a^	1886.19 ± 112.01^c^	4215.14 ± 106.26^b^

Note: Experimental data are expressed as mean ± standard error. Different lowercase letters indicate significant differences between groups (*P* < 0.05). ND indicates not detected in the sample.

Acetaldehyde was the predominant carbonyl compound in all four treatments, accounting for 42.66%–61.18% of the total aldehyde and ketone concentrations. Short-chain carbonyl compounds (C_1_–C_3_) generally have low boiling points and high vapor pressures, which promote their volatilization and partitioning into the gas phase during high-temperature cooking (Xiang et al., 2017). These compounds can also arise from the cleavage of saturated fatty acids and oxidative scission of unsaturated fatty acids. Therefore, their multiple formation pathways may account for their predominance in the carbonyl profiles. Crotonaldehyde and methacrolein were not detected under these experimental conditions. As reactive unsaturated aldehydes, these compounds may undergo polymerization or further oxidation in a heated, enclosed chamber. The acrolein/acetone analyte was detected only in the OO and CO groups. In both treatments, the fume exhaust remained open during the residual heat phase, suggesting that the exhaust state during this phase may have influenced the persistence or analytical recovery of acrolein/acetone.

### 3.7 Correlation analysis between aldehydes and ketones in cooking fumes and volatile flavor compounds in pork belly

OPLS-DA was used to characterize the treatment-dependent differences in the volatile profiles of pork belly. The associations of pork belly volatiles with fume-derived aldehydes and ketones were examined using correlation network analysis. As shown in Fig. 5A, replicate samples within each treatment formed relatively compact clusters, indicating low variability within the group. The four treatment groups were clearly separated in the score plot, demonstrating distinct volatile profiles (Cai et al., 2016). Model quality assessment revealed that the OPLS-DA model showed a high goodness-of-fit (R^2^Y = 0.995) and favorable predictive capability (Q^2^ = 0.965). The robustness of the model was further validated through permutation testing (*n* = 200), with no evidence of overfitting observed, thereby confirming the reliability of the multivariate analysis results (Fig. 5B).

**Fig. 5:**
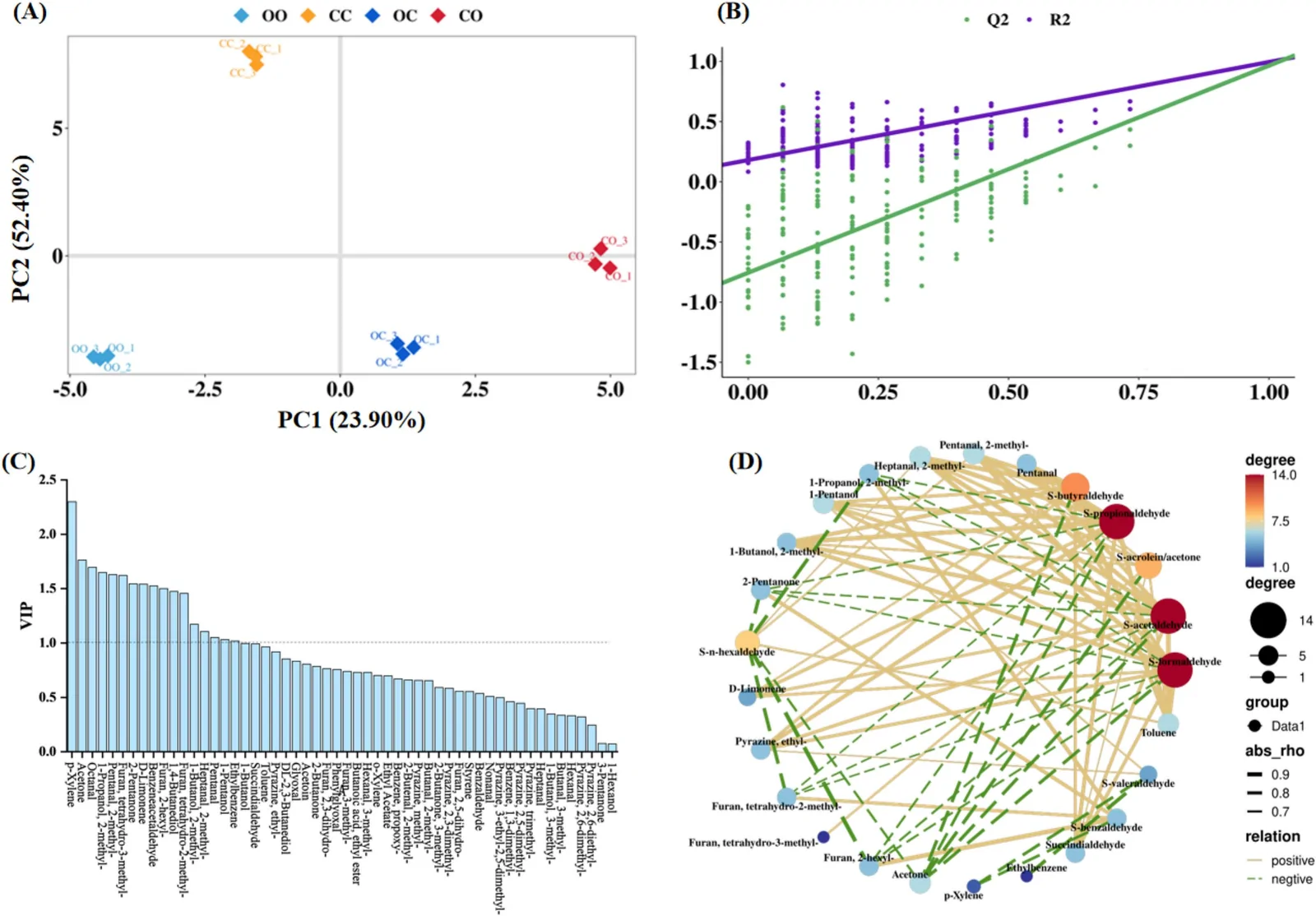
(A) Scatter plot of carbonyl compounds and volatile compounds in cooking fumes; (B) substitution test plot; (C) VIP values; (D) correlation network analysis.

Variable importance in projection (VIP) scores were used to rank the contribution of individual compounds to group discrimination. Variables with VIP scores >1.0 were considered influential discriminators (Chen et al., 2011). As illustrated in Fig. 5C, 17 volatile compounds in pork belly exceeded this threshold. These compounds comprised the alkylbenzenes p-xylene and ethylbenzene; the terpene d-limonene; the aldehydes octanal, pentanal, 2-methylpentanal, 2-methylheptanal, and phenylacetaldehyde; the ketones acetone and 2-pentanone; the alcohols 2-methyl-1-propanol, 1,4-butanediol, 2-methyl-1-butanol, and 1-pentanol; and the furans tetrahydro-3-methylfuran, 2-hexylfuran, and tetrahydro-2-methylfuran. A correlation network was constructed of the pork belly volatiles with the aldehydes and ketones detected in the cooking fumes (Fig. 5D). The prefix “S-” was used to distinguish fume-derived carbonyls from volatile compounds detected in pork belly. Topological analysis indicated that S-formaldehyde, S-acetaldehyde, S-acrolein/acetone, S-propionaldehyde, and S-butyraldehyde had the highest node degrees, demonstrating that these carbonyls were the most highly connected variables within the network. Positive associations connected these hub nodes with succindialdehyde, pentanal, 2-methylpentanal, 2-methylheptanal, 2-methyl-1-propanol, 1-pentanol, 2-methyl-1-butanol, and toluene. These relationships indicate that the corresponding compounds tended to vary in the same direction across treatments. In contrast, these hub carbonyls were negatively associated with acetone, 2-hexylfuran, and tetrahydro-2-methylfuran, indicating an inverse treatment-dependent relationship.

Overall, this correlation network reveals complex interactions among volatile compounds in the thermal processing system. Positive correlations predominate, suggesting that the majority of flavor compounds tend to be generated synergistically, whereas negative correlations may reflect mechanisms such as substrate competition or pathway diversion. Fume-derived aldehydes and ketones, serving as core nodes of the network, not only exerted regulatory effects on the overall network structure but also potentially influenced the generation and accumulation of characteristic flavor substances such as furans and other ketones through competition for common precursors or interference with reaction pathways.

## 4 Conclusion

This study systematically elucidated the association between pork belly flavor development and cooking oil fume emissions under different fume exhaust modes in an intelligent stir-frying system. Keeping the fume exhaust closed throughout cooking (CC) promoted the accumulation of characteristic volatile compounds, particularly pyrazines and furans. Odor activity value analysis identified 3-methylbutanal, 2-methylbutanal, hexanal, and 2,5-dimethylpyrazine as the principal aroma contributors, with these compounds exhibiting their highest OAVs in the CC group. Enhanced flavor development in the CC mode was associated with increased thermal degradation of proteins in a high-fume-concentration environment. Accordingly, the CC group exhibited the highest total free amino acid content (760.99 mg/100 g). Among the individual amino acids, Glu and Ala had taste activity values exceeding 1, indicating substantial contributions to umami and sweetness. Conversely, the high-fume-concentration environment was associated with a pronounced reduction in the fatty acid content, particularly that of polyunsaturated fatty acids. Despite its favorable flavor profile, the CC mode generated the highest total aldehyde and ketone concentration, reaching 4868.89 μg/m^3^. Acetaldehyde was the predominant component, accounting for 42.66%–61.18% of the total aldehydes and ketones, indicating a potential health concern. Correlation network analysis showed that fume-derived formaldehyde and acetaldehyde were positively associated with pentanal and 2-methyl-1-butanol in pork belly but negatively associated with 2-hexylfuran levels. These relationships suggest that synergistic formation and competitive interactions may occur between fume-derived aldehydes and ketones and meat-derived flavor compounds, potentially through substrate competition or shifts in reaction pathways. Overall, closing the fume exhaust during heating and opening it during the residual heat phase (CO) provided the most favorable balance between flavor quality and emission control. This mode maintained a comparatively high flavor quality while decreasing total aldehyde and ketone emissions by approximately 13.4% relative to the CC mode. These findings provide practical guidance for optimizing intelligent stir-frying processes and developing adaptive fume-control strategies that preserve desirable sensory properties while reducing potentially harmful emissions.

## CRediT authorship contribution statement

**Jingnan Lu:** Writing – original draft, Methodology, Investigation, Funding acquisition. **Honglie Ding:** Writing – original draft, Methodology, Investigation. **Jiajin Sun:** Methodology. **Lizhang Wu:** Methodology. **Manzi Hu:** Methodology. **Kang Xu:** Methodology. **Huimin Wang:** Methodology. **Xingguang Chen:** Writing – review & editing, Supervision, Funding acquisition. **Kezhou Cai:** Writing – review & editing, Supervision, Methodology, Funding acquisition. **Baocai Xu:** Writing – review & editing, Supervision.

## Declaration of competing interest

The authors declare that they have no known competing financial interests or personal relationships that could have appeared to influence the work reported in this paper.

## Data Availability

Data will be made available on request.
