# Supplementary material for: Revealing the mechanistic correlation between volatile flavor retention and cooking oil fume reduction in stir-fried pork belly
Source: Food Chem X. 2026 Sep 30;39:104547. doi: 10.1016/j.fochx.2026.104547 (PMC13658858; doi:10.1016/j.fochx.2026.104547)
Supplement: Supplementary file 1 [file mmc1.docx]

**Revealing the mechanistic correlation between volatile flavor retention and cooking oil fume reduction in stir-fried pork belly**

Jingnan Lu ^a^, Honglie Ding^a^, Jiajin Sun^a^, Lizhang Wu^a^, Manzi Hu^a^, Kang Xu^a^, Huimin Wang^c^, Xingguang Chen^a^, Kezhou Cai ^a,b,*^, Baocai Xu ^a,b^

^a^ *Key Laboratory for Animal Food Green Manufacturing and Resource Mining of Anhui Province, Hefei University of Technology, Hefei 230009, China*

^b^ *School of Food and Bioengineering, Hefei University of Technology, Hefei 230009, China*

^c^Graduate Institute of Biomedical Engineering, National Chung Hsing University, Taichung City 402, Taiwan, China

E-*mail address:* 183487484@qq.com (Jingnan Lu)

*E-mail address:* 1617023723@qq.com (Honglie Ding)

*E-mail address:* sjj15957801615@163.com (Jiajin Sun)

*E-mail address:* 1872492869@qq.com (Lizhang Wu)

*E-mail address:* 2487315209@qq.com (Manzi Hu)

*E-mail address:* 2511910946@qq.com (Kang Xu)

*E-mail address:*davidw@dragon.nchu.edu.tw (Huimin Wang)

*E-mail address:* 2738480412@qq.com (Xingguang Chen)

** E-mail address:* kzcai@hfut.edu.cn (Kezhou Cai)

*E-mail address:* baocaixu@163.com (Baocai Xu)

* Corresponding author

Kezhou Cai, Ph.D./Professor, Engineering Research Center of Bio-process, Hefei University of Technology, 420 Feicui Road, Hefei 230601, Anhui Province, PRC.

Tel.: +86 15305516697; Fax: +86 551 62901507.

Table S1 Volatile flavor compounds identified in pork belly by GC-MS analysis

| Compound | RI | Identification | | CAS No. | | Formula | | Content (μg/kg) | | | | | | | |
| --- | --- | --- | --- | --- | --- | --- | --- | --- | --- | --- | --- | --- | --- | --- | --- |
|  |  |  |  |  |  |  |  | OO | | CC | | OC | | CO | |
| Glyoxal |  | MS, LRI | | 107-22-2 | | C_2_H_2_O_2_ | | ND | | 1.18±0.21^a^ | | ND | | 0.28±0.04^b^ | |
| 3-Methylbutanal | 547 | MS, LRI | | 590-86-3 | | C_5_H_10_O | | 1.12±0.35^c^ | | 5.53±0.13^a^ | | 1.39±0.4^c^ | | 2.72±0.17^b^ | |
| 2-Methylbutanal | 544 | MS, LRI | | 96-17-3 | | C_5_H_10_O | | 0.66±0.2^c^ | | 7.36±1.34^a^ | | 1.50±0.25^c^ | | 3.98±0.43^b^ | |
| Succinaldehyde | 567 | MS, LRI | | 638-37-9 | | C_4_H_6_O_2_ | | ND | | 3.23±0.68^a^ | | ND | | 0.94±0.06^b^ | |
| Pentanal | 571 | MS, LRI | | 110-62-3 | | C_5_H_10_O | | ND | | 3.58±0.48^a^ | | ND | | 1.37±0.41^b^ | |
| 2-Methylpentanal | 616 | MS, LRI | | 123-15-9 | | C_6_H_12_O | | ND | | 4.04±0.84^a^ | | 1.00±0.18^b^ | | 1.93±0.47^b^ | |
| 2-Methylheptanal | 635 | MS, LRI | | 16630-91-4 | | C_8_H_16_O | | ND | | 2.69±0.46^a^ | | ND | | 1.55±0.36^b^ | |
| Hexanal | 772 | MS, LRI | | 66-25-1 | | C_6_H_12_O | | 11.72±1.22^c^ | | 22.76±0.89^a^ | | 16.27±1.1^b^ | | 18.51±1.78^b^ | |
| Heptanal | 867 | MS, LRI | | 111-71-7 | | C_7_H_14_O | | 0.54±0.18^c^ | | 2.06±0.05^a^ | | 0.74±0.08^c^ | | 1.35±0.44^b^ | |
| 3-Methylhexanal | 868 | MS, LRI | | 19269-28-4 | | C_7_H_14_O | | ND | | 0.94±0.07 | | ND | | ND | |
| Benzaldehyde | 923 | MS, LRI | | 100-52-7 | | C_7_H_6_O | | 0.73±0.06^c^ | | 4.09±0.48^a^ | | 1.46±0.45^c^ | | 2.64±0.76^b^ | |
| Phenylglyoxal | 924 | MS, LRI | | 1074-12-0 | | C_8_H_6_O_2_ | | ND | | 1.45±0.15 | | ND | | ND | |
| Octanal | 957 | MS, LRI | | 124-13-0 | | C_8_H_16_O | | 1.16±0.25^c^ | | 2.41±0.18^a^ | | 1.49±0.33^b^ | | ND | |
| 2-Methyl-2-butenal | 957 | MS, LRI | | 1115-11-3 | | C_5_H_8_O | | ND | | 0.37±0.17 | | ND | | ND | |
| Phenylacetaldehyde | 995 | MS, LRI | | 122-78-1 | | C_8_H_8_O | | 0.57±0.09^c^ | | 2.55±0.16^a^ | | 1.42±0.29^b^ | | ND | |
| Nonanal | 1042 | MS, LRI | | 124-19-6 | | C_9_H_18_O | | 1.52±0.41^d^ | | 7.56±0.2^a^ | | 3.32±0.53^c^ | | 4.45±0.35^b^ | |
| Aldehydes (total) |  |  | |  | |  | | 18.02±1.41^d^ | | 71.80±4.77^a^ | | 28.60±1.37^c^ | | 39.72±2.66^b^ | |
| Ethyl acetate |  | MS, LRI | | 141-78-6 | | C_4_H_8_O_2_ | | 1.68±0.33^d^ | | 9.22±0.47^a^ | | 4.85±0.26^c^ | | 6.87±0.36^b^ | |
| Ethyl butanoate | 770 | MS, LRI | | 105-54-4 | | C_6_H_12_O_2_ | | 2.04±0.08^b^ | | 11.68±1.66^a^ | | 3.68±0.71^b^ | | 0.66±0.29^c^ | |
| Esters (total) |  |  | |  | |  | | 3.73±0.29^c^ | | 20.91±2.08^a^ | | 8.53±0.94^b^ | | 7.53±0.26^b^ | |
| 2-Methyl-1-propanol | 793 | MS, LRI | | 78-83-1 | | C_4_H_10_O | | 0.95±0.56 | | ND | | ND | | ND | |
| Acetoin | 682 | MS, LRI | | 513-86-0 | | C_4_H_8_O_2_ | | 4.53±0.6^b^ | | ND | | 3.40±1.49^b^ | | 4.87±0.85^a^ | |
| 1-Pentanol | 703 | MS, LRI | | 71-41-0 | | C_5_H_12_O | | ND | | 5.36±1.02^a^ | | ND | | 1.46±0.37^b^ | |
| 3-Methyl-1-butanol | 708 | MS, LRI | | 123-51-3 | | C_5_H_12_O | | 5.74±0.2^c^ | | 21.97±1.29^a^ | | 6.83±0.55^c^ | | 13.37±1.73^b^ | |
| 2-Methyl-1-butanol | 712 | MS, LRI | | 137-32-6 | | C_5_H_12_O | | ND | | 11.59±0.37^a^ | | ND | | 4.64±1.54^b^ | |
| DL-2,3-Butanediol | 772 | MS, LRI | | 6982-25-8 | | C_4_H_10_O_2_ | | ND | | 7.45±0.59 | | ND | | ND | |
| 1-Hexanol | 841 | MS, LRI | | 111-27-3 | | C_6_H_14_O | | ND | | 0.69±0.02^b^ | | 1.60±0.66^a^ | | ND | |
| 1-Butanol | 946 | MS, LRI | | 71-36-3 | | C_4_H_10_O | | ND | | ND | | 1.18±0.57^a^ | | 0.04±0^b^ | |
| 1,4-Butanediol | 1050 | MS, LRI | | 110-63-4 | | C_4_H_10_O_2_ | | 0.36±0.13^c^ | | 0.61±0.06^a^ | | 0.56±0.29^b^ | | ND | |
| Alcohols (total) |  |  | |  | |  | | 11.58±1.21^c^ | | 47.66±2.55^a^ | | 13.57±1.20^c^ | | 24.38±3.29^b^ | |
| Acetone |  | MS, LRI | | 67-64-1 | | C_3_H_6_O | | 2.03±0.74^a^ | | 0.58±0.05^b^ | | 1.43±0.84^ab^ | | ND | |
| 3-Pentanone |  | MS, LRI | | 96-22-0 | | C_5_H_10_O | | ND | | 0.93±0.32^a^ | | 0.78±0.21^b^ | | ND | |
| 2-Butanone |  | MS, LRI | | 78-93-3 | | C_4_H_8_O | | ND | | ND | | 5.45±0.86 | | ND | |
| 3-Methyl-2-butanone | 669 | MS, LRI | | 563-80-4 | | C_5_H_10_O | | ND | | ND | | 0.22±0.03 | | ND | |
| 2-Pentanone | 631 | MS, LRI | | 107-87-9 | | C_5_H_10_O | | 0.50±0.3 | | ND | | ND | | ND | |
| Ketones (total) |  |  | |  | |  | | 2.52±0.58^b^ | | 1.51±0.34^b^ | | 7.88±0.73^a^ | | ND | |
| Toluene | 740 | MS, LRI | | 108-88-3 | | C_7_H_8_ | | 0.31±0.11^d^ | | 10.75±1.23^a^ | | 2.02±0.06^c^ | | 5.60±0.73^b^ | |
| Propoxybenzene | 809 | MS, LRI | | 622-85-5 | | C_9_H_12_O | | ND | | 0.39±0.08 | | ND | | ND | |
| o-Xylene | 839 | | MS, LRI | | 95-47-6 | | C_8_H_10_ | | 3.29±2.42^a^ | | 2.42±1.28^b^ | | 3.19±0.98^a^ | | 0.71±0.16^c^ |
| p-Xylene | 840 | | MS, LRI | | 106-42-3 | | C_8_H_10_ | | ND | | ND | | 1.97±0.13^b^ | | 2.72±0.41^a^ |
| 1,3-Dimethylbenzene | 841 | | MS, LRI | | 108-38-3 | | C_8_H_10_ | | 0.67±0.12^c^ | | 4.16±1.19^a^ | | 1.33±0.41^bc^ | | 2.08±0.15^b^ |
| Ethylbenzene | 841 | | MS, LRI | | 100-41-4 | | C_8_H_10_ | | 1.01±0.21^b^ | | ND | | 2.43±0.57^a^ | | 2.66±0.41^a^ |
| Styrene | 860 | | MS, LRI | | 100-42-5 | | C_8_H_8_ | | 0.67±0.27^c^ | | 5.27±1.23^a^ | | 1.82±0.16^bc^ | | 2.45±0.22^b^ |
| D-Limonene | 981 | | MS, LRI | | 5989-27-5 | | C_10_H_16_ | | ND | | 2.40±1.06^a^ | | 0.77±0.12^b^ | | 1.10±0.52^ab^ |
| Hydrocarbons (total) |  | |  | |  | |  | | 5.95±2.07^d^ | | 25.40±1.42^a^ | | 13.53±1.78^c^ | | 17.33±0.60^b^ |
| Methylpyrazine | 800 | | MS, LRI | | 109-08-0 | | C_5_H_6_N_2_ | | 1.40±0.44^b^ | | 14.29±6.37^a^ | | 3.75±1.18^b^ | | 7.60±0.59^b^ |
| 2,5-Dimethylpyrazine | 879 | | MS, LRI | | 123-32-0 | | C_6_H_8_N_2_ | | 2.18±1.34^c^ | | 8.22±1.07^a^ | | 3.07±0.52^c^ | | 5.44±0.6^b^ |
| 2,6-Dimethylpyrazine | 881 | | MS, LRI | | 108-50-9 | | C_6_H_8_N_2_ | | 0.63±0.26^b^ | | 2.72±0.65^a^ | | 0.93±0.21^b^ | | 1.37±0.4^b^ |
| Ethylpyrazine | 884 | | MS, LRI | | 13925-00-3 | | C_6_H_8_N_2_ | | ND | | 1.98±0.47^a^ | | ND | | 0.52±0.06^b^ |
| 2,3-Dimethylpyrazine | 889 | | MS, LRI | | 5910-89-4 | | C_6_H_8_N_2_ | | 0.31±0.08^b^ | | 1.33±0.31^a^ | | 0.43±0.09^b^ | | 1.19±0.2^a^ |
| Trimethylpyrazine | 959 | | MS, LRI | | 14667-55-1 | | C_7_H_10_N_2_ | | 1.00±0.39^b^ | | 4.06±1.75^a^ | | 1.37±0.46^b^ | | 2.48±0.32^ab^ |
| 3-Ethyl-2,5-dimethylpyrazine | 1024 | | MS, LRI | | 13360-65-1 | | C_8_H_12_N_2_ | | 1.07±0.02^a^ | | 0.58±0.05^ab^ | | 1.06±0.44^a^ | | 0.49±0.32^b^ |
| 2,6-Diethylpyrazine | 1024 | | MS, LRI | | 13067-27-1 | | C_8_H_12_N_2_ | | 0.64±0.45^b^ | | 5.03±0.88^a^ | | 1.06±0.25^b^ | | 0.70±0.39^b^ |
| Pyrazines (total) |  | |  | |  | |  | | 7.23±1.53^c^ | | 38.20±7.07^a^ | | 11.67±2.34^c^ | | 19.78±1.19^b^ |
| Tetrahydro-2-methylfuran | 631 | | MS, LRI | | 96-47-9 | | C_5_H_10_O | | 0.29±0.18 | | ND | | ND | | ND |
| Tetrahydro-3-methylfuran | 568 | | MS, LRI | | 13423-15-9 | | C_5_H_10_O | | ND | | ND | | ND | | 0.69±0.15 |
| 3-Methylfuran | 768 | | MS, LRI | | 930-27-8 | | C_5_H_6_O | | ND | | 1.17±0.47 | | ND | | ND |
| 2-Hexylfuran | 882 | | MS, LRI | | 3777-70-6 | | C_10_H_16_O | | 0.34±0.15 | | ND | | ND | | ND |
| 2,3-Dihydrofuran | 776 | | MS, LRI | | 1191-99-7 | | C_4_H_6_O | | 0.26±0.07^a^ | | 0.12±0.03^b^ | | ND | | 0.12±0.05^b^ |
| 2,5-Dihydrofuran | 703 | | MS, LRI | | 1708-29-8 | | C_4_H_6_O | | ND | | ND | | 0.15±0.06 | | ND |
| Furans (total) |  | |  | |  | |  | | 0.89±0.14^a^ | | 1.28±0.50^a^ | | 0.15±0.06^b^ | | 0.81±0.12^a^ |
| Grand total |  | |  | |  | |  | | 49.91±3.86^d^ | | 206.75±16.37^a^ | | 83.92±4.67^c^ | | 109.54±6.90^b^ |

Table S2 Qualitative analysis of VOCs in stir-fried pork belly by GC-IMS

| No. | Compound | CAS | Formula | MW | RI | RT (s) | Dt (a.u) |
| --- | --- | --- | --- | --- | --- | --- | --- |
| 1 | Methyl furoate | C611132 | C6H6O3 | 126.1 | 1587.8 | 1465.681 | 1.15298 |
| 2 | Benzaldehyde | C100527 | C7H6O | 106.1 | 1534.6 | 1247.235 | 1.15866 |
| 3 | Acetic acid-M | C64197 | C2H4O2 | 60.1 | 1489.9 | 1088.802 | 1.05762 |
| 4 | Acetic acid-D | C64197 | C2H4O2 | 60.1 | 1491.3 | 1093.603 | 1.16206 |
| 5 | 2-Ethylpyrazine | C13925003 | C6H8N2 | 108.1 | 1394.1 | 813.944 | 1.16206 |
| 6 | ( E)-2-octenal | C2548870 | C8H14O | 126.2 | 1437.7 | 929.168 | 1.34371 |
| 7 | 1-nonanal | C124196 | C9H18O | 142.2 | 1404.6 | 840.349 | 1.48789 |
| 8 | 1 -hexanol | C111273 | C6H14O | 102.2 | 1369.4 | 755.131 | 1.33803 |
| 9 | Ethyl 2-hydroxypropanoate | C97643 | C5H10O3 | 118.1 | 1358.8 | 731.126 | 1.14957 |
| 10 | 2,3-Dimethylpyrazine | C5910894 | C6H8N2 | 108.1 | 1367.5 | 750.806 | 1.11518 |
| 11 | Furan, 2-methyl-3-(methylthio) | C63012975 | C6H8OS | 128.2 | 1350.7 | 713.465 | 1.10991 |
| 12 | Ethyl heptanoate | C106309 | C9H18O2 | 158.2 | 1340.9 | 692.572 | 1.41985 |
| 13 | (E)-2-Heptenal-M | C18829555 | C7H12O | 112.2 | 1331.7 | 673.377 | 1.26505 |
| 14 | (E)-2-Heptenal-D | C18829555 | C7H12O | 112.2 | 1332 | 673.996 | 1.6889 |
| 15 | 2-Butanone, 3-hydroxy-M | C513860 | C4H8O2 | 88.1 | 1299.2 | 610.218 | 1.06357 |
| 16 | 2-Butanone, 3-hydroxy-D | C513860 | C4H8O2 | 88.1 | 1297.6 | 607.122 | 1.34122 |
| 17 | 1-Pentanol-M | C71410 | C5H12O | 88.1 | 1262.5 | 552.014 | 1.26137 |
| 18 | 1-Pentanol-D | C71410 | C5H12O | 88.1 | 1262.1 | 551.395 | 1.52796 |
| 19 | 2-Ethoxyethanol | C110805 | C4H10O2 | 90.1 | 1253.7 | 539.011 | 1.10411 |
| 20 | 2-pentyl furan | C3777693 | C9H14O | 138.2 | 1240.6 | 520.435 | 1.26628 |
| 21 | ( E)-2-hexen-1-al | C6728263 | C6H10O | 98.1 | 1228.5 | 503.716 | 1.18888 |
| 22 | Butanoic acid butyl ester-M | C109217 | C8H16O2 | 144.2 | 1226.6 | 501.239 | 1.35351 |
| 23 | Butanoic acid butyl ester-D | C109217 | C8H16O2 | 144.2 | 1225.7 | 500.001 | 1.85229 |
| 24 | 1-Butanol, 3-methyl-M | C123513 | C5H12O | 88.1 | 1217.8 | 489.475 | 1.25277 |
| 25 | 1-Butanol, 3-methyl-D | C123513 | C5H12O | 88.1 | 1216.9 | 488.236 | 1.5083 |
| 26 | Heptaldehyde-M | C111717 | C7H14O | 114.2 | 1197 | 462.849 | 1.34122 |
| 27 | Heptaldehyde-D | C111717 | C7H14O | 114.2 | 1196.5 | 462.23 | 1.71838 |
| 28 | Butanoicac id propyl ester-M | C105668 | C7H14O2 | 130.2 | 1186.7 | 450.522 | 1.27124 |
| 29 | Butanoicac id propyl ester-D | C105668 | C7H14O2 | 130.2 | 1186.1 | 449.892 | 1.7094 |
| 30 | 1- butanol-M | C71363 | C4H10O | 74.1 | 1155.2 | 415.833 | 1.18746 |
| 31 | 1- butanol-D | C71363 | C4H10O | 74.1 | 1155.2 | 415.833 | 1.39239 |
| 32 | Butyl propanoate-M | C590012 | C7H14O2 | 130.2 | 1149.5 | 409.842 | 1.29615 |
| 33 | Butyl propanoate-D | C590012 | C7H14O2 | 130.2 | 1151.3 | 411.734 | 1.74676 |
| 34 | 1-Propanol, 2-methyl-M | C78831 | C4H10O | 74.1 | 1105.7 | 366.638 | 1.1784 |
| 35 | 1-Propanol, 2-methyl-D | C78831 | C4H10O | 74.1 | 1104.7 | 365.692 | 1.37314 |
| 36 | 1-hexanal-M | C66251 | C6H12O | 100.2 | 1098.5 | 360.016 | 1.26671 |
| 37 | 1-hexanal-D | C66251 | C6H12O | 100.2 | 1097.5 | 359.07 | 1.57919 |
| 38 | 2-Hexanone | C591786 | C6H12O | 100.2 | 1097.5 | 359.07 | 1.54636 |
| 39 | Acetic acid butyl ester-M | C123864 | C6H12O2 | 116.2 | 1084.9 | 349.294 | 1.24747 |
| 40 | Acetic acid butyl ester-D | C123864 | C6H12O2 | 116.2 | 1084.5 | 348.978 | 1.64033 |
| 41 | 3-Methyl-2-butenal | C107868 | C5H8O | 84.1 | 1211.9 | 481.742 | 1.09802 |
| 42 | 1-Propanol-M | C71238 | C3H8O | 60.1 | 1050.9 | 324.381 | 1.115 |
| 43 | 1-Propanol-D | C71238 | C3H8O | 60.1 | 1050 | 323.75 | 1.26219 |
| 44 | Butanoic acid ethyl ester-M | C105544 | C6H12O2 | 116.2 | 1049.6 | 323.435 | 1.21463 |
| 45 | Butanoic acid ethyl ester-D | C105544 | C6H12O2 | 116.2 | 1050 | 323.75 | 1.57806 |
| 46 | ID_1 | unidentified | * | 0 | 1001.9 | 291.584 | 1.30181 |
| 47 | 2-Pentanone | C107879 | C5H10O | 86.1 | 997.1 | 288.542 | 1.38157 |
| 48 | ID_2 | unidentified | * | 0 | 997.6 | 288.861 | 1.40891 |
| 49 | n-Pentanal | C110623 | C5H10O | 86.1 | 1000.2 | 290.456 | 1.43814 |
| 50 | ID_3 | unidentified | * | 0 | 968.3 | 275.145 | 1.7295 |
| 51 | Ethanol | C64175 | C2H6O | 46.1 | 941.8 | 263.503 | 1.13264 |
| 52 | 2-methyl butanal-M | C96173 | C5H10O | 86.1 | 921.8 | 255.05 | 1.1647 |
| 53 | 2-methyl butanal-D | C96173 | C5H10O | 86.1 | 924.9 | 256.326 | 1.41646 |
| 54 | 2-Butanone-M | C78933 | C4H8O | 72.1 | 912.2 | 251.063 | 1.06381 |
| 55 | 2-Butanone-D | C78933 | C4H8O | 72.1 | 913 | 251.382 | 1.25805 |
| 56 | Acetic acid ethyl ester-M | C141786 | C4H8O2 | 88.1 | 897.6 | 245.162 | 1.10247 |
| 57 | Acetic acid ethyl ester-D | C141786 | C4H8O2 | 88.1 | 896 | 244.524 | 1.35045 |
| 58 | Butanal-M | C123728 | C4H8O | 72.1 | 886.3 | 240.696 | 1.11944 |
| 59 | Butanal-D | C123728 | C4H8O | 72.1 | 887.5 | 241.175 | 1.29388 |
| 60 | 2-propanone | C67641 | C3H6O | 58.1 | 841.7 | 223.791 | 1.12322 |
| 61 | 1-octene | C111660 | C8H16 | 112.2 | 849.9 | 226.821 | 1.16659 |
| 62 | Propanal-M | C123386 | C3H6O | 58.1 | 825.3 | 217.89 | 1.05816 |
| 63 | Propanal-D | C123386 | C3H6O | 58.1 | 824.4 | 217.571 | 1.15433 |
| 64 | ID_4 | unidentified | * | 0 | 834.2 | 221.079 | 1.29388 |
| 65 | 2-Heptanone | C110430 | C7H14O | 114.2 | 1193.3 | 458.24 | 1.67955 |
| 66 | isoamyl formate-M | C110452 | C6H12O2 | 116.2 | 1079.1 | 344.951 | 1.27494 |
| 67 | isoamyl formate-D | C110452 | C6H12O2 | 116.2 | 1079.4 | 345.139 | 1.67162 |
| 68 | 2-Methyl butanoic acid ethyl ester-M | C7452791 | C7H14O2 | 130.2 | 1063.6 | 333.498 | 1.25084 |
| 69 | 2-Methyl butanoic acid ethyl ester-D | C7452791 | C7H14O2 | 130.2 | 1063.4 | 333.31 | 1.67464 |
| 70 | ID_5 | unidentified | * | 0 | 866 | 232.855 | 1.09518 |
